# Effects of Heat Waves on Hospitalizations, Emergency Department Visits, and Outpatient Care in Frail Older Adults: A Systematic Review and Meta-Analysis

**DOI:** 10.3390/diseases14050176

**Published:** 2026-05-18

**Authors:** Antonio Pinto, Flavia Pennisi, Stefania Borlini, Emanuele De Ponti, Carlo Signorelli, Andrea Cozza, Vincenzo Baldo, Vincenza Gianfredi

**Affiliations:** 1 Faculty of Medicine, University Vita-Salute San Raffaele, 20132 Milan, Italy; pinto.antonio@hsr.it (A.P.); sborlini@asst-pg23.it (S.B.); deponti.emanuele@hsr.it (E.D.P.); signorelli.carlo@hsr.it (C.S.); 2 Ph.D. National Program in One Health Approaches to Infectious Diseases and Life Science Research, Department of Public Health, Experimental and Forensic Medicine, University of Pavia, 27100 Pavia, Italy; 3 Department of Cardiac Thoracic Vascular Sciences and Public Health, University of Padua, 35128 Padova, Italy; andrea.cozza@unipd.it (A.C.); vincenzo.baldo@unipd.it (V.B.)

**Keywords:** heat waves, frailty, older adults, healthcare utilization, hospitalizations, emergency department visits, outpatient care, climate change, public health

## Abstract

Background/Objectives: Heat waves are increasingly frequent and intense climate events with significant implications for public health, particularly among frail older adults. While most evidence has focused on mortality and morbidity, healthcare service utilization represents an additional and potentially more sensitive indicator of heat-related health burden. Methods: A systematic review and meta-analysis was conducted following the PRISMA guidelines and prospectively registered in PROSPERO (CRD420251107598). PubMed/MEDLINE, Scopus, and Web of Science were searched up to August 2025. This study aimed to systematically review and quantitatively synthesize the evidence on the association between heat wave exposure and healthcare utilization—including hospitalizations, emergency department (ED) visits, and outpatient care—among frail older adults. Pooled effect estimates (RRs, IRRs, and ORs) were calculated using random-effects models. Heterogeneity was assessed using the I^2^ statistic, and sensitivity analyses were performed by outcome type, effect measure, and risk of bias. Results: Fifty-five studies met the inclusion criteria. Heat wave exposure was consistently associated with increased healthcare utilization. Both hospitalizations and ED visits showed significant increases during heat wave periods, with results remaining robust across sensitivity analyses. Evidence on outpatient care was limited but suggested a similar pattern. Substantial heterogeneity was observed across studies, reflecting variability in exposure definitions, populations, and study designs. Overall, the methodological quality of the included studies was acceptable, with most presenting a low-to-moderate risk of bias. Conclusions: Heat waves are associated with increased healthcare utilization among frail older adults, indicating a relevant burden on healthcare systems. Healthcare utilization may represent a sensitive indicator of heat wave impact, complementing traditional clinical outcomes.

## 1. Introduction

Heat waves represent one of the most significant extreme climate events affecting public health, as well as social life, the environment, and overall well-being [1,2]. In the context of climate change, they are becoming increasingly frequent, more intense, and longer lasting [3,4]. According to a 2022 study [5], it is estimated that more than 60,000 deaths in Europe alone were attributable to heat-related stress.

Among the groups at highest risk are frail older adults, due to the combined effects of age-related physiological changes, the presence of comorbidities, reduced thermoregulatory capacity, chronic dehydration, the potential use of medications that interfere with hydro-electrolytic homeostasis, and social factors such as isolation and barriers to accessing protective resources, including air conditioning and care services [6,7,8,9,10]. In this context, beyond clinical outcomes, it is crucial to consider the impact of heat waves on healthcare utilization, as it reflects both the burden of disease and the pressure exerted on healthcare systems [11,12].

Despite the extensive literature on the health effects of extreme heat, the comparability of evidence on heat waves is limited by the heterogeneity of definitions used and by the frequent terminological overlap between “heat waves” and single days of extreme heat [13]. As heat waves typically consist of multi-day, consecutive events, the present review aims to synthesize the evidence by focusing on exposures that reflect this episodic nature and the associated strain on healthcare services. Accordingly, this systematic review and meta-analysis seeks to critically summarize the available evidence on the effects of heat waves on healthcare utilization, such as hospitalizations, emergency department visits, and outpatient care, among frail older adults aged ≥ 60 years and to explore potential differences according to population characteristics (e.g., sex) and geographic context.

## 2. Materials and Methods

This systematic review and meta-analysis was conducted and reported in accordance with the Preferred Reporting Items for Systematic Reviews and Meta-Analyses (PRISMA 2020) statement. The review protocol was developed a priori and registered in PROSPERO (CRD420251107598). No major amendments to the review methods were made after registration.

### 2.1. Research Strategy

A systematic search was conducted in PubMed/MEDLINE, Scopus, and Web of Science up to August 2025. There were no time restrictions; articles published in English or Italian were eligible for inclusion. The full electronic search strategies for all databases are reported in Supplementary Table S1.

The primary search strategy for PubMed was constructed by combining MeSH terms and free-text keywords related to: (i) climate change/extreme heat/heat waves, (ii) healthcare utilization (hospitalizations, emergency department visits, and outpatient care), and (iii) older adults/frailty.

Specific search strings were tailored to each database. Additional records were identified through reference list screening and consultation with experts/authors where applicable.

### 2.2. Inclusion Criteria for Studies

The inclusion/exclusion criteria were defined according to the PECOS scheme:

- Population (P): Frail elderly individuals (≥60 years). Studies analyzing subgroups of frail or vulnerable older adults according to the definition adopted by the authors (e.g., clinical/care frailty, multimorbidity, or vulnerability conditions explained in the study) were included.
- Exposure (E): Heat waves. Studies evaluating periods identified as “heat wave/heat waves” by the authors were included, with the operational definition (e.g., percentile/absolute threshold) and temporal structure of the episode reported. For the purposes of this review, episodes were considered consistent with the concept of heat wave if they included consecutive periods of elevated temperature lasting several days, as characterized in the international literature [14,15,16] and by authoritative meteorological and public health organizations, including the World Meteorological Organization (WMO) [17], the World Health Organization (WHO) [18], and the United Nations Office for Disaster Risk Reduction (UNDRR) [19]. Individual days of extreme heat or non-consecutive hot days were considered not aligned with the exposure of interest and were excluded.
- Comparator (C): Non-heat wave periods/reference conditions defined by the study (e.g., control days in case-crossover study design; pre–post periods; exposed vs. unexposed regions).
- Outcome (O): Use of healthcare systems: (i) hospitalizations, (ii) emergency department visits, and (iii) outpatient care. Effects (ORs/RRs/IRRs/MDs with 95% CIs) were extracted when available.
- Study design (S): Non-randomized observational studies, including prospective and retrospective cohort studies, case–control studies, cross-sectional studies, time-series analyses, case-crossover studies, and other relevant observational designs. Editorials and commentaries, reviews, qualitative studies, and modeling studies without individual- or population-level observational data suitable for exposure–outcome association analyses, as well as studies lacking specific data on older or frail populations or without healthcare utilization outcomes, were excluded.

### 2.3. Study Selection

All records retrieved were imported into reference management web software (Rayyan, Rayyan Systems Inc., Broadway, Cambridge, MA, USA), and duplicates were removed. After duplicate removal, two reviewers independently performed: (i) title and abstract screening and (ii) full-text assessment of potentially eligible articles. Discrepancies were resolved through consultation with a third reviewer. Inclusion and exclusion decisions, along with reasons for exclusion, were documented in a dedicated screening management file.

### 2.4. Data Extraction

Two reviewers independently extracted data using a standardized form, pre-piloted on 2 randomly selected included studies. For each included study, the following variables were extracted:

- Study characteristics: author (year of publication), study period, study design, country, level of data analysis (national, regional, provincial, city, multi-level, or hospital/health facility), number of participants, age, percentage of females, and population characteristics.
- Exposure (heat waves): exposure assessment method, heat wave definition, any intensity index, season and exposure period, lag structure, meteorological variables considered, baseline period, and type of comparison (e.g., pre–post, across regions, and control days).
- Outcomes (healthcare utilization): outcome assessment method, unit of analysis, type of outcome (hospitalizations, emergency department visits, or outpatient care), and specific health event.
- Statistical analysis: statistical methods, effect measure and maximally adjusted estimate with 95% confidence interval (CI), type of effect measure (OR, RR, IRR, or MD), and covariates included in the adjustment.
- Additional information: conflicts of interest and funding sources.

When multiple estimates were reported for the same outcome, the most fully adjusted estimate was selected for the primary synthesis. When information was unclear or incomplete, data were extracted conservatively from the published report.

### 2.5. Data Synthesis and Statistical Analysis

A meta-analysis was performed to pool data and estimate the association between heat wave exposure and healthcare utilization outcomes across studies. Studies were included in the quantitative synthesis when they reported an effect estimate for the association between heat wave exposure and at least one healthcare utilization outcome in older adults, together with sufficient information to derive variance estimates. Studies that did not provide sufficient quantitative information were retained for qualitative synthesis only. For each study, the most fully adjusted effect estimate for the heat wave–outcome association was extracted. Relative effect measures, including risk ratios (RRs), incidence rate ratios (IRRs), and odds ratios (ORs), together with their 95% confidence intervals (CIs), were used as the common metrics. Because these are multiplicative measures with approximately log-normal distributions, all effect estimates were analyzed on the logarithmic scale using an inverse-variance method. Standard errors (SEs) were derived from the reported 95% CIs, and pooled estimates were back-transformed to the ratio scale for interpretation.

Random-effects models were used as the primary analytical approach to account for between-study heterogeneity due to differences in study design, heat wave definitions, exposure metrics, lag structures, outcome ascertainment, and geographic context. Fixed-effect models were computed only as supplementary analyses. Each study contributed one independent estimate per outcome to the primary model. When multiple lag periods, exposure definitions, or model specifications were reported within the same study, all eligible effect estimates were extracted and included as separate entries in the meta-analysis. These estimates were treated as distinct effect sizes (ESs) to fully capture variability related to lag structure and model specification.

Heterogeneity was assessed using the I^2^ statistic and interpreted as follows: not important (I^2^ < 25%), low (25% ≤ I^2^ < 50%), moderate (50% ≤ I^2^ < 75%), or high (I^2^ ≥ 75%) [20]. Publication bias was evaluated through visual inspection of funnel plots and Egger’s tests, with a *p*-value of <0.10 indicating the presence of bias [21]. In cases where publication bias was detected, the trim-and-fill method was applied to adjust for potentially missing studies.

All statistical analyses were conducted using STATA version 18 (StataCorp LLC, 4905 Lakeway Drive, College Station, TX, USA) and Prometa3^®^ software (Internovi, Cesena, Italy).

Individual study results were summarized in structured tables, and quantitative syntheses were displayed using forest plots; funnel plots were used to explore small-study effects.

### 2.6. Sensitivity Analyses

A series of prespecified sensitivity analyses were conducted to evaluate the robustness of the pooled estimates and to explore potential sources of heterogeneity. First, analyses were stratified by outcome type, with separate models restricted to hospitalizations only and ED visits only, in order to assess the consistency of the association across different healthcare utilization endpoints. Second, sensitivity analyses were conducted according to the type of reported effect measure. Separate meta-analyses were performed, including only studies reporting ORs and, independently, only studies reporting RRs/IRRs. These restricted models were compared with the main pooled analyses to evaluate the consistency of the association across different effect measure types. Third, methodological robustness was examined by repeating the meta-analyses after exclusion of studies judged to be at high risk of bias according to the predefined risk of bias assessment tool.

### 2.7. Risk of Bias Assessment

Risk of bias was independently assessed by two reviewers, with disagreements resolved by consensus or by consultation with a third reviewer. In accordance with the heterogeneity of the included study designs, two different assessment tools were applied depending on study type. For observational analytical studies conducted at the individual level, including case-crossover, self-controlled case series, and matched or time-stratified designs, risk of bias was assessed using the Risk Of Bias In Non-randomized Studies of Interventions (ROBINS-I) tool [22]. The assessment addressed bias due to confounding, participant selection, exposure classification, deviations from intended exposures, missing data, outcome measurement, and selective reporting. For studies based on aggregated or population-level data, such as ecological, descriptive, and time-series designs, the Office of Health Assessment and Translation (OHAT) risk of bias tool [23] was applied. OHAT was chosen because it is specifically designed for observational environmental and population-based studies and allows a domain-based assessment when exposure and outcome are measured at the group level. The OHAT assessment covered domains related to selection bias, confounding, attrition/exclusion bias, exposure characterization, outcome assessment, selective reporting, and other sources of bias, with an overall evaluation of confidence in the body of evidence. In addition, the overall certainty of the evidence was judged narratively for each main outcome domain (hospitalizations, emergency department visits, and outpatient care) by considering study limitations, inconsistency, indirectness, imprecision, and possible publication bias.

## 3. Results

### 3.1. Literature Search

**Figure 1:**
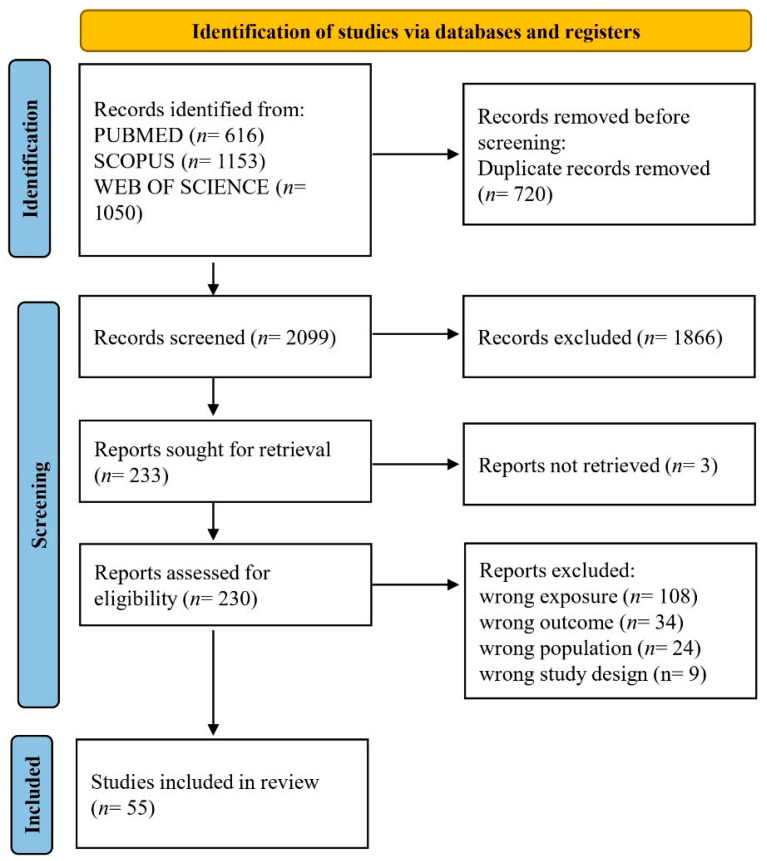
PRISMA 2020 flow diagram of the study selection process for the systematic review and meta-analysis.

A total of 2819 records were identified by searching PubMed/MEDLINE (*n* = 616), Scopus (*n* = 1153), and Web of Science (*n* = 1050). No additional articles were included based on reference screening and expert consultation. After the preliminary exclusion of duplicates (*n* = 720), a total of 2099 records were screened based on title and abstract. Based on the initial screening, records were excluded due to their being conference papers (*n* = 39), being written in different languages (*n* = 71), or focusing on unrelated topics or populations (*n* = 1756), resulting in 233 records deemed eligible for inclusion. Three papers were excluded because the full texts could not be retrieved. Based on the full-text assessment of the 230 records, the following were excluded: *n* = 108 due to wrong exposure, *n* = 34 due to wrong outcome, *n* = 24 due to wrong population, and *n* = 9 due to wrong study design, resulting in 55 records included in the current systematic review. The study selection process is presented in the PRISMA 2020 flow diagram (Figure 1).

### 3.2. Geographical Distribution

**Figure 2:**
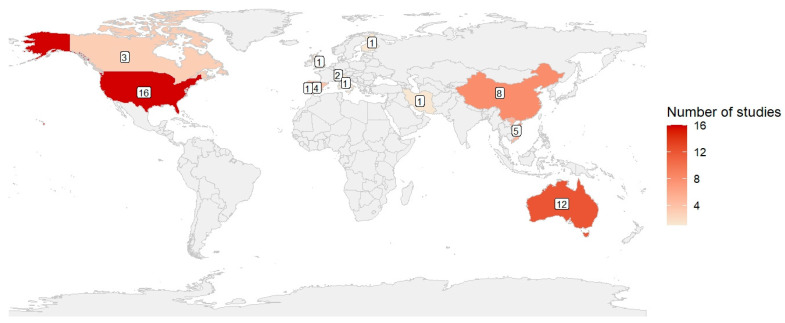
Geographical distribution of studies by country.

The geographical distribution of the included studies is shown in Figure 2. Overall, the evidence was unevenly distributed across countries, with a marked concentration of studies conducted in high-income settings. At the country level, the largest number of studies was identified in the United States (*n* = 16) [24,25,26,27,28,29,30,31,32,33,34,35,36,37,38,39], followed by Australia (*n* = 12) [40,41,42,43,44,45,46,47,48,49,50,51] and China (*n* = 8) [52,53,54,55,56,57,58,59]. A smaller but non-negligible number of studies was conducted in Vietnam (*n* = 5) [60,61,62,63,64], Spain (*n* = 4) [65,66,67,68], and Canada (*n* = 3) [69,70,71]. Switzerland contributed two studies [72,73], while Iran [74], Portugal [75], Italy [76], the United Kingdom [77], and Finland [78] were each represented by a single study. Overall, most of the available evidence originated from North America, East and Southeast Asia, Europe, and Oceania, whereas large regions such as Africa and South America were not represented. No studies including multiple countries were identified; therefore, all the studies included could be attributed to a specific national context.

### 3.3. Study Characteristics

Ecological time-series-based analyses represented the most frequently adopted methodological approach (*n* = 30) [26,27,30,32,34,35,38,39,40,41,42,47,48,49,52,56,57,58,61,62,63,64,65,66,67,72,74,75,76,78], encompassing standard, retrospective, and matched ecological time-series designs. These studies were primarily aimed at estimating short-term population-level associations between heat waves and healthcare utilization. Case-crossover designs were also widely used (*n* = 13) [24,28,29,31,36,46,50,51,52,53,54,59,60], most commonly implemented through time-stratified or symmetric bidirectional approaches. These quasi-experimental methods enabled within-individual comparisons over time, thereby reducing confounding by time-invariant individual characteristics. Event-based, population-level, or self-controlled ecological case-series approaches accounted for six studies [43,44,45,68,71,73], while the remaining studies employed other observational designs (*n* = 6) [25,33,37,69,70,77], including descriptive ecological analyses, and quasi-experimental observational studies.

Most analyses were conducted at the city level (*n* = 19) [25,34,37,39,40,41,45,48,49,50,52,54,55,57,58,59,62,63], followed by regional-level, national-level, and hospital/health facility-based analyses, all of which were covered by 10 papers. Fewer studies were conducted at the provincial level (*n* = 5) [56,60,64,67,71], while only one study [53] adopted a multi-level analytical approach combining different geographic or administrative scales.

The included studies covered a wide temporal span, with study periods ranging from the early 1970s to the early 2020s. The earliest observations began in 1973 [72], whereas the most recent studies included data up to 2021 [52,66,70]. Most investigations were based on extended observation windows, often exceeding 10 years, allowing for the assessment of heat wave effects across multiple warm seasons and climatic contexts. The majority of studies focused on periods between the mid-1990s and the late 2010s, reflecting the increasing availability of routinely collected health and meteorological data in recent decades.

Most studies analyzed all-age populations while reporting results for older subgroups defined according to age thresholds or age strata. The most commonly used lower age cut-off to define older populations was 65 years (*n* = 44) [24,25,26,27,28,29,30,31,32,33,34,35,36,37,38,39,40,41,42,44,45,46,48,49,50,51,53,54,55,59,61,62,65,66,67,68,69,71,72,73,74,75,77,78], although some studies adopted alternative thresholds, including ≥60 (*n* = 7) [52,56,57,58,60,63,64], ≥70 (*n* = 1) [43], or ≥75 (*n* = 2) [47,76] years. A substantial number of studies (*n* = 34) [24,25,26,27,28,30,33,34,35,37,38,39,40,41,42,44,45,46,47,48,50,54,55,60,62,64,67,69,73,74,75,76,77,78] did not report sex-specific descriptive statistics, limiting comparability across studies. Among the studies reporting sex distribution, the proportion of women ranged from 23.9% [63] to 66% [32], with the majority of study populations comprising more than 50% female participants.

The main characteristics of the included studies are summarized in Table 1.

### 3.4. Operational Characteristics of Heat Wave Exposure Definitions

Marked heterogeneity was observed in the operational definitions of heat waves across the included studies (Table 2). Heat wave exposure was most commonly defined using relative, percentile-based temperature thresholds (*n* = 37) [24,26,28,29,30,31,32,35,36,38,39,40,42,46,48,49,50,51,52,53,57,58,60,61,62,63,64,65,67,68,69,70,71,72,74,75,78], primarily based on daily mean (Tmean) or maximum temperature (Tmax). Percentile cut-offs ranged from the 90th to the 99th percentile, with the 95th percentile being the most frequently applied threshold (*n* = 20) [24,25,28,29,39,40,42,48,49,50,52,58,60,65,67,68,71,72,74,78].

The minimum duration required to define a heat wave varied from one to seven consecutive days. Definitions based on at least two consecutive days (*n* = 23) [25,26,28,29,30,31,34,35,36,38,39,46,48,49,51,52,53,58,61,62,65,66,72] of threshold exceedance were the most commonly adopted, followed by definitions requiring three consecutive days (*n* = 18) [32,41,42,43,45,54,55,56,57,59,63,64,68,69,70,71,75,76]. Some studies (*n* = 3) [40,50,74] evaluated multiple duration thresholds within the same analysis.

Several studies (*n* = 15) [25,27,34,40,42,43,44,48,52,54,57,59,63,65,76] adopted absolute temperature thresholds, either alone or in combination with percentile-based criteria. These definitions typically relied on fixed cut-offs expressed in degrees Celsius or Fahrenheit, most often based on Tmax or Tmean.

In addition to threshold–duration criteria, a limited number of studies explicitly incorporated heat wave intensity or severity indices. These approaches included the use of the Excess Heat Factor (EHF) (*n* = 3) [38,50,75], apparent temperature-based metrics such as the heat index (*n* = 3) [25,27,37], and percentile-based intensity classifications (*n* = 8) [24,28,36,48,49,62,63,78]. However, the majority of studies (*n* = 38) [26,30,31,32,33,34,35,39,40,41,42,43,44,45,46,47,51,52,53,54,55,56,57,59,60,61,64,65,66,67,68,69,70,71,72,73,74,77] did not report a distinct intensity index, and heat wave exposure was defined solely on the basis of temperature threshold exceedance and minimum duration.

Most studies assessed heat wave exposure during the summer or warm season (*n* = 47) [24,25,27,28,29,31,32,33,34,35,36,37,39,40,41,42,43,44,45,46,47,48,49,50,52,53,54,55,56,57,58,59,60,63,65,67,68,69,70,71,72,73,74,75,76,77,78], while a smaller subset (*n* = 8) [26,30,38,51,61,62,64,66] considered all-year exposure windows.

Lag structures showed substantial variability across studies. Most analyses evaluated short-term effects, with lag windows ranging from same-day exposure (lag 0) to cumulative periods of up to 7 days. The maximum lag was the 21-day evaluation. Distributed lag non-linear models (DLNMs) (*n* = 8) [39,53,57,59,63,65,66,67] were sometimes applied to characterize delayed and cumulative effects across multiple lag days. A substantial number of studies (*n* = 22) [25,27,30,31,32,33,34,35,36,41,42,43,44,45,51,68,69,70,73,75,76,77] did not explicitly report the lag structure.

Regarding the comparison framework, most studies (*n* = 48) [6,7,8,9,10,11,12,13,14,15,16,17,18,19,20,21,22,23,24,25,26,27,28,29,30,31,32,33,34,35,36,37,38,39,40,41,42,43,44,45,68,71,72,74,75,76,77,78] compared heat wave days with non-heat wave days within the same location. Alternative approaches were used in a smaller number of studies.

#### Meteorological Variables

**Figure 3:**
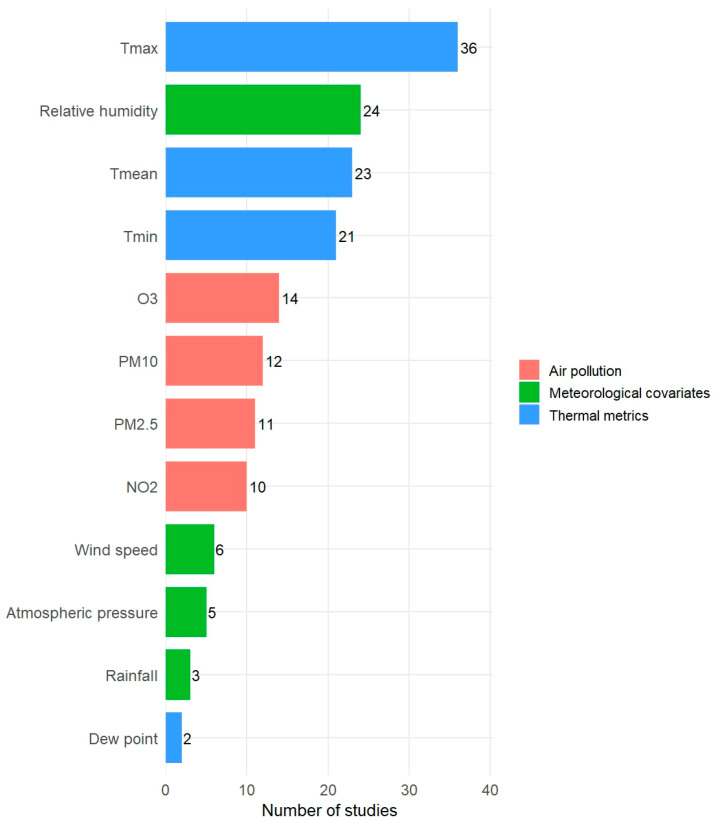
Meteorological variables used in included studies.

Across studies, heat wave exposure assessment was frequently accompanied by the inclusion of additional meteorological and environmental variables (Figure 3). Thermal metrics were the most commonly used variables, with daily maximum temperature (Tmax) included in the largest number of studies (*n* = 36) [25,31,32,33,34,36,39,40,41,42,43,44,45,46,47,48,49,50,52,54,55,56,60,61,63,64,66,67,68,69,70,71,73,74,75,76], followed by daily mean temperature (Tmean) (*n* = 23) [24,26,28,29,30,35,38,39,40,46,49,51,53,55,56,58,60,61,62,65,66,74,78], daily minimum temperature (Tmin) (*n* = 21) [33,34,39,40,42,43,44,46,47,49,52,54,60,61,66,67,69,70,73,74,75], and dew point (*n* = 2) [28,31]. Air pollution variables were incorporated in a subset of studies, most commonly ozone (O_3_) (*n* = 14) [25,29,31,38,39,46,48,52,53,54,65,66,72,78], PM_10_ (*n* = 12) [29,38,46,48,49,50,54,65,66,72,74,78], PM_2.5_ (*n* = 11) [25,31,38,50,51,52,53,58,65,74,78], and NO_2_ (*n* = 10) [31,38,46,48,49,50,54,65,72,78]. Among meteorological covariates, relative humidity was the most frequently considered variable (*n* = 24) [29,31,38,48,49,50,52,53,54,55,56,57,58,59,60,61,62,63,64,65,72,74,76,78], whereas wind speed (*n* = 6) [38,50,53,55,57,59], atmospheric pressure (*n* = 5) [52,55,56,57,72], and rainfall (*n* = 3) [59,64,72] were included less consistently.

### 3.5. Healthcare Utilization Outcomes

As shown in Table 3, across the included studies, heat wave exposure was examined in relation to a wide range of healthcare utilization outcomes, encompassing hospitalizations (*n* = 32) [24,25,26,28,29,30,31,34,35,37,38,41,42,46,50,53,54,55,56,57,60,61,62,63,64,67,69,72,74,75,76,78], emergency department (ED) visits (*n* = 16) [27,36,39,40,44,45,47,48,49,51,65,66,69,70,73,77], and outpatient care (*n* = 2) [58,59]. Five studies evaluated mixed outcomes: specifically, four [32,33,43,71] assessed hospitalizations combined with emergency department visits and one [52] examined outpatient care together with emergency department visits.

Outcomes were predominantly evaluated using daily aggregated counts of healthcare events (*n* = 42) [25,26,27,28,30,32,33,34,35,37,38,39,40,41,42,43,44,45,47,48,49,51,52,53,54,56,57,58,61,62,63,64,65,66,67,69,71,72,73,74,75,76,78], although several studies employed individual-level data (*n* = 13) [24,29,31,36,46,50,55,59,60,68,69,71,77]. The majority of investigations assessed all-cause healthcare utilization (*n* = 25) [25,26,27,28,30,31,32,33,34,35,36,39,43,44,47,48,49,51,57,65,66,69,73,75,76], while others focused on cause-specific outcomes, including cardiovascular, respiratory, renal, neurological, metabolic, and heat-related conditions. Some studies evaluated multiple disease categories or specific diagnoses within the same analysis.

#### Analytical Approaches and Outcome Metrics

Across studies, statistical methods varied according to study design and outcome data structure. Most analyses were based on count regression models applied to daily aggregated outcomes, including Poisson or quasi-Poisson generalized linear models, negative binomial models, and related extensions within GAM/GLM frameworks to account for seasonality, long-term trends, and calendar effects (*n* = 25) [32,35,39,41,42,43,45,47,48,49,51,56,58,61,62,64,65,66,67,69,71,72,73,76,78]. A substantial subset of studies used time-stratified case-crossover designs, typically implemented through conditional logistic regression or conditional Poisson regression, enabling within-unit comparisons between heat wave and non-heat wave periods defined by the matching strategy (*n* = 16) [24,28,29,31,36,38,46,50,51,53,54,55,59,60,68,71]. Lagged and cumulative effects were addressed heterogeneously: in studies explicitly modeling distributed lag structures, exposure–response associations were estimated using distributed lag non-linear models (DLNM) (*n* = 8) [39,53,56,61,65,67,72,74] or distributed lag models (DLM) (*n* = 3) [5,52,57]. In contrast, several studies evaluated pre-specified single-day lags or cumulative windows without specifying a distributed lag framework.

Effect estimates were reported using heterogeneous metrics, most commonly RRs (*n* = 28) [26,28,30,31,33,34,35,39,47,48,49,52,53,54,56,57,58,59,61,62,63,64,65,66,67,72,73,78], IRRs (*n* = 9) [32,40,41,42,45,68,75,76,77], or ORs (*n* = 10) [24,29,36,38,46,50,51,55,60,71], whereas a smaller number of studies reported absolute excess counts, DIDs, CERs, mortality rates, or percentage changes (*n* = 8) [25,27,37,43,44,69,70,74].

### 3.6. Direction and Consistency of Reported Associations

Most studies (*n* = 48) [28,29,30,31,32,33,34,35,36,37,38,39,40,41,42,43,44,45,47,48,49,50,51,53,54,55,56,57,58,59,60,61,62,63,64,65,66,69,70,71,72,73,74,75,76,78] reported a positive association between heat wave exposure and healthcare utilization among older adults, indicating increased risks of hospital admissions, ED visits, or outpatient encounters during heat wave periods. Several studies reported null or weak associations for specific outcomes, age groups, or lag periods, and a limited number of analyses observed inverse or non-significant effects (*n* = 7) [24,25,46,52,67,68,77].

However, the magnitude and statistical significance of reported associations varied substantially across studies, outcome definitions, and analytical approaches.

### 3.7. Meta-Analysis

The overall quantitative synthesis, including all eligible effect estimates, demonstrated a statistically significant association between heat wave exposure and healthcare utilization outcomes. Effect estimates were pooled on the logarithmic scale using inverse-variance weighting. Under the random-effects model, the pooled ES was 0.18 (95% CI 0.15–0.20), with high heterogeneity (I^2^ = 97.4%, *p* < 0.001). Back-transformation to the ratio scale corresponds to a pooled relative effect of approximately 1.20 (95% CI 1.16–1.22). Under the fixed-effect model, the pooled ES was 0.14 (95% CI 0.12–0.16), corresponding to a back-transformed relative effect of approximately 1.15 (95% CI 1.13–1.17). Visual analysis of the funnel plot and Egger’s test (intercept: 3.07, *p* < 0.001) confirmed potential publication bias.

In sensitivity analyses excluding studies judged to be at high risk of bias, the association remained statistically significant and directionally consistent with the primary analysis. Using the restricted dataset, the pooled effect estimate under the random-effects model, the pooled effect estimate was 0.15 (95% CI 0.13–0.17), corresponding to a back-transformed relative effect of approximately 1.16 (95% CI 1.14–1.19). Under the fixed-effect model, the pooled effect estimate was 0.05 (95% CI 0.04–0.06), corresponding to a back-transformed relative effect of approximately 1.05 (95% CI 1.04–1.06).

**Figure 4:**
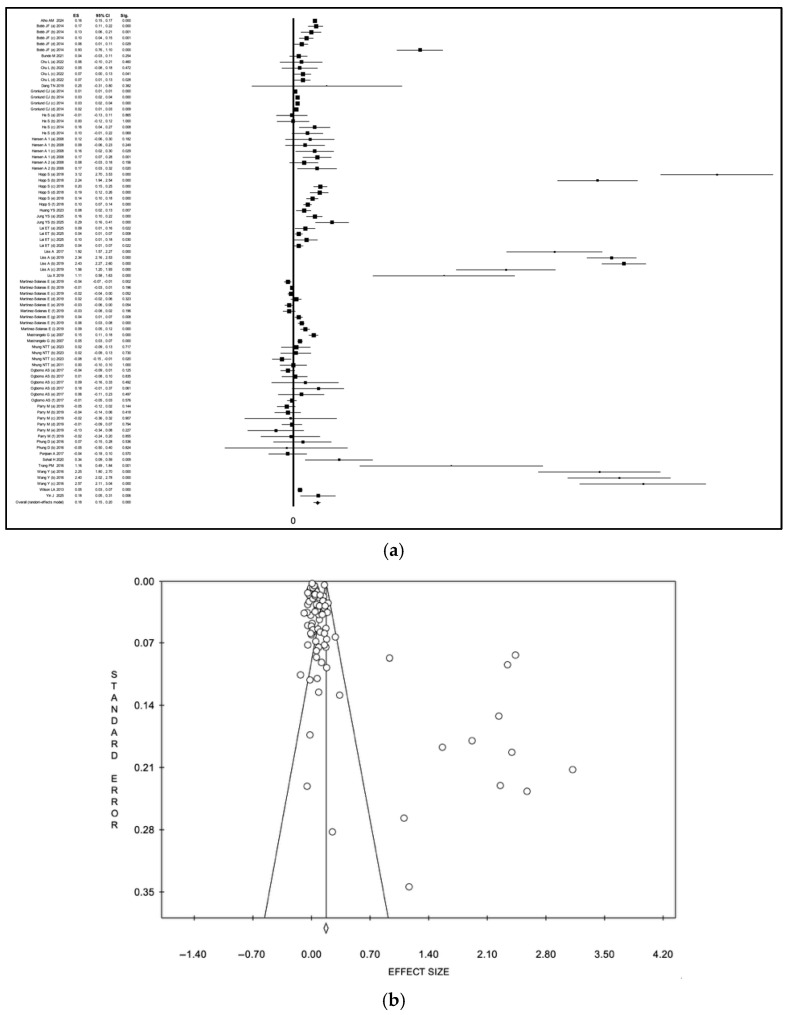
(**a**) A forest plot and (**b**) funnel plot of the random-effects model assessing hospitalizations.

In the meta-analysis restricted to hospitalization outcomes, pooled estimates remained positive, although model-dependent differences in magnitude were observed. Using an inverse-variance random-effects model [24,26,28,29,30,31,34,38,41,42,46,49,50,52,54,55,56,60,62,64,67,68,75,76,78], the pooled logarithmic ES was substantially larger at 0.18 (95% CI 0.15–0.20), corresponding to a back-transformed relative effect of approximately 1.20 (95% CI 1.16–1.22). Under the fixed-effect model, the pooled logarithmic ES was 0.04 (95% CI 0.04–0.05), corresponding to a back-transformed relative effect of approximately 1.04–1.05, indicating a modest increase in hospitalizations during heat wave periods (Figure S1, a: forest plot; b: funnel plot). Heterogeneity remained high (I^2^ = 97.9%, *p* < 0.001). These results are presented in Figure 4 (a: forest plot; b: funnel plot). Visual analysis of the funnel plot and Egger’s test (intercept: 3.22, *p* < 0.001) confirmed potential publication bias.

**Figure 5:**
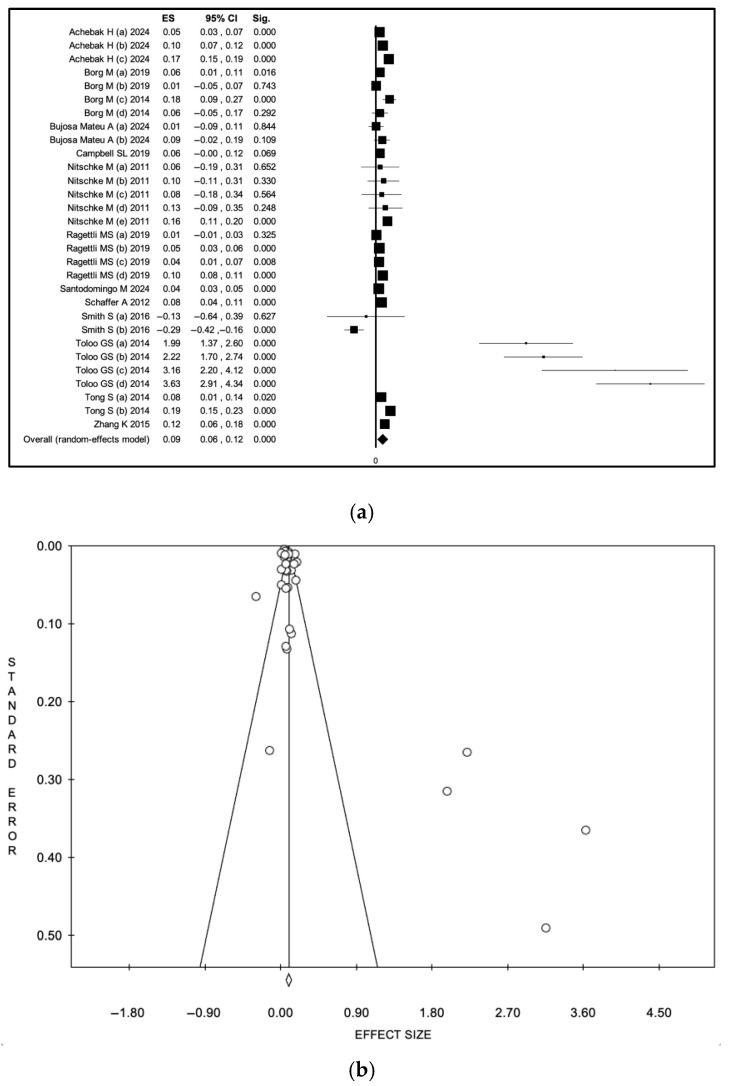
(**a**) A forest plot and (**b**) funnel plot of the random-effects model assessing emergency department visits.

In analyses restricted to ED visits, heat wave exposure was associated with a statistically significant increase in event rates across studies. Using an inverse-variance random-effects model [36,39,40,45,47,48,49,51,65,66,73,77], the pooled logarithmic ES was 0.09 (95% CI 0.06–0.12), corresponding to a back-transformed relative effect of approximately 1.09 (95% CI 1.06–1.13). Under the fixed-effect model, the pooled logarithmic ES was 0.06 (95% CI 0.06–0.07), corresponding after back-transformation to a relative effect of approximately 1.06–1.07 (Figure S2, a: forest plot; b: funnel plot; Figure 5, a: forest plot; b: funnel plot). Heterogeneity remained high (I^2^ = 94.4%, *p* < 0.001). Visual analysis of the funnel plot and Egger’s test (intercept: 2.44, *p* 0.02) confirmed potential publication bias.

**Figure 6:**
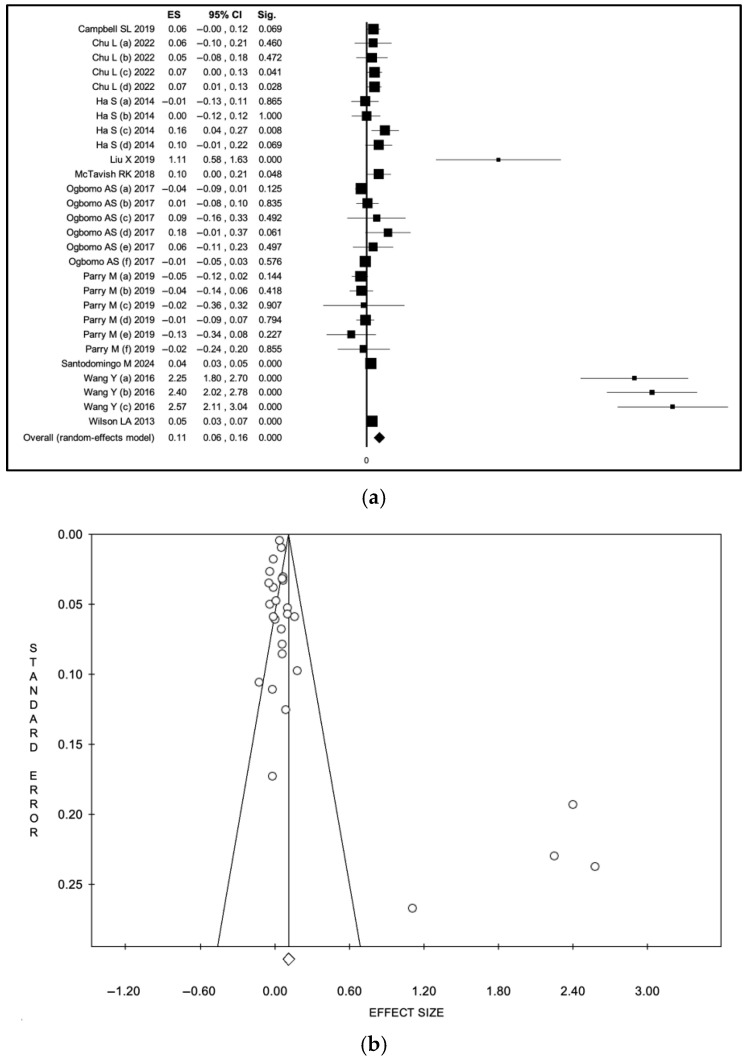
(**a**) A forest plot and (**b**) funnel plot of the random-effects model assessing studies reporting odds ratios.

In sensitivity analyses restricted to studies reporting ORs, the association between heat wave exposure and healthcare utilization remained positive, although the magnitude of the pooled effect varied by the meta-analytic model. Using a random-effects model [24,29,36,38,46,51,55,60,71], the pooled logarithmic effect size increased to 0.11 (95% CI 0.06–0.16), corresponding to a back-transformed odds ratio of approximately 1.12 (95% CI 1.06–1.17). Under the fixed-effect model, the pooled logarithmic ES was 0.04 (95% CI 0.03–0.05), corresponding after back-transformation to an overall odds ratio of approximately 1.04 (95% CI 1.03–1.05) (Figure S3, a: forest plot; b: funnel plot; Figure 6, a: forest plot; b: funnel plot). Heterogeneity remained high (I^2^ = 93.5%, *p* < 0.001). Visual analysis of the funnel plot and Egger’s test (intercept: 2.41, *p* 0.02) confirmed potential publication bias.

**Figure 7:**
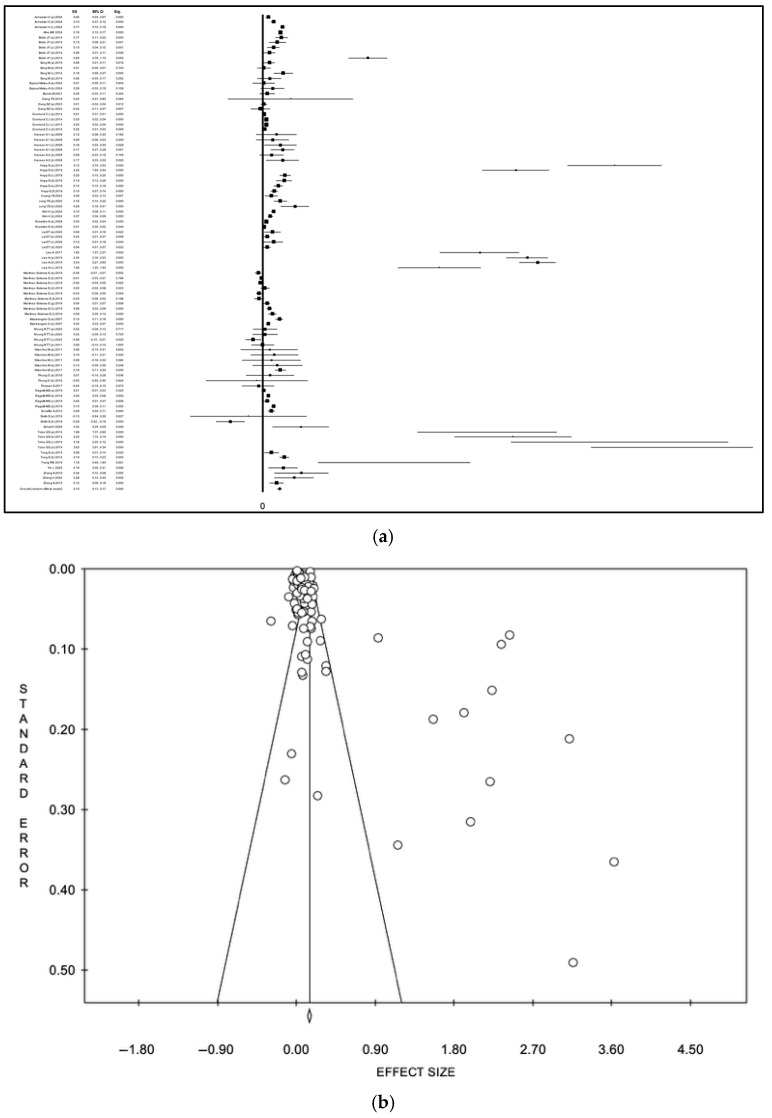
(**a**) A forest plot and (**b**) funnel plot of the random-effects model assessing studies reporting risk ratios or incidence rate ratios.

In sensitivity analyses restricted to studies reporting RRs or IRRs, the association between heat wave exposure and healthcare utilization remained statistically significant. Using a random-effects model [26,28,30,31,32,33,34,35,40,41,42,45,47,52,54,56,58,61,62,63,64,65,66,67,68,72,73,75,76,77], the pooled logarithmic effect size increased to 0.15 (95% CI 0.13–0.17), corresponding to a back-transformed relative effect of approximately 1.16 (95% CI 1.14–1.19) (Figure 7, a: forest plot; b: funnel plot). Under the fixed-effect model, the pooled logarithmic effect size was 0.05 (95% CI 0.04–0.06), corresponding after back-transformation to a pooled relative effect of approximately 1.05 (95% CI 1.04–1.06) (Figure S4, a: forest plot; b: funnel plot) Heterogeneity remained high (I^2^ = 97.8%, *p* < 0.001). Visual analysis of the funnel plot and Egger’s test (intercept: 3.63, *p* < 0.001) confirmed potential publication bias.

### 3.8. Risk of Bias Assessment

**Figure 8:**
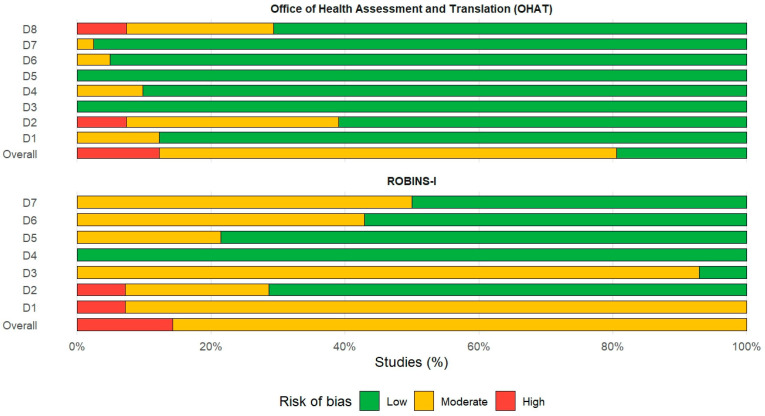
Distribution of risk of bias assessments across the eight domains of the Office of Health Assessment and Translation (OHAT) tool and the seven domains of the ROBINS-I instrument.

The results of the risk of bias assessment are presented in Figure 8. According to the OHAT assessment, most population-level and ecological studies were judged to have low-to-moderate overall risk of bias (8 low [26,30,38,48,49,56,57,65] and 27 moderate [25,27,28,32,34,35,37,39,40,41,42,45,47,52,58,61,62,63,64,66,67,72,73,74,75,76,78] out of 41 studies). Across domains, selection bias, attrition/exclusion bias, exposure characterization, outcome assessment, selective reporting, and other sources of bias were generally rated as low or probably low risk. In contrast, confounding and overall confidence in the body of evidence represented the most frequent sources of concern. A smaller subset of studies, mainly descriptive or event-based ecological designs, were judged to be at high or definitely high risk of bias (five high [33,43,53,69,77] and one [44] definitely high), largely due to limitations in confounding control and reduced confidence in the body of evidence. Using the ROBINS-I tool, most individual-level observational studies (12 [24,29,31,36,46,50,51,54,55,59,60,68] out of 14) were judged to be at moderate overall risk of bias, reflecting primarily concerns related to residual confounding and exposure classification. The domains most frequently rated as moderate were bias due to confounding, exposure classification and bias in selection of the reported result, whereas bias related to selection of participants, deviations from intended exposures, missing data, and outcome measurement was generally assessed as low. Two studies [70,71] were judged to be at serious overall risk of bias, driven mainly by serious concerns in participant selection and confounding. No study was rated to be at critical risk of bias.

## 4. Discussion

### 4.1. Summary of Results

The meta-analysis demonstrated a statistically significant association between heat wave exposure and increased healthcare utilization. The results remained robust and consistent across all sensitivity analyses, including those restricted to studies at low risk of bias, therefore reinforcing the strength of the observed association.

Both hospitalizations and emergency department visits increased significantly during heat wave periods. Stratified analyses by effect measure type further confirmed the positive direction of the association, with largely concordant estimates.

Heterogeneity across studies was high in all analyses, suggesting considerable variability in study designs, populations, and definitions of heat waves. The overall methodological quality of the included studies was acceptable, with most studies presenting a low-to-moderate risk of bias. The main concerns were related to confounding control and the overall confidence in the body of evidence. No study was rated to be at critical risk of bias.

### 4.2. Interpretation of Main Findings

The analysis highlights the considerable public health relevance of these findings within the current epidemiological and environmental context. Although the observed risk increases may appear modest in relative terms, their impact at the population level is substantial, particularly given the presence of systemic vulnerability factors such as population aging [79], increasing urbanization, and the already strained capacity of healthcare systems [80,81].

To facilitate the interpretation of ratio measures, the pooled estimates can be translated into approximate absolute numbers under explicit assumptions. For example, in a hypothetical European city of 1,000,000 inhabitants, assuming that 22% of residents are aged ≥65 years, in line with recent Eurostat estimates for the European Union, the older population would include approximately 220,000 individuals [82]. If the baseline rate of ED presentations among older adults were assumed to be 0.5 per 1000 persons per day, a value within the range of annual ED utilization reported in European and OECD settings, approximately 110 ED presentations would be expected each day among older adults under non-heat wave conditions. Applying the pooled relative effect for ED visits observed in the present meta-analysis, approximately 1.09, would correspond to about 10 additional ED presentations per day, or approximately 70 additional ED presentations during a 7-day heat wave episode. Assuming that 20–28% of ED visits result in hospital admission, as reported in OECD and European emergency care statistics, this could translate into roughly 14–20 additional hospital admissions over the same period [83]. This example is illustrative only and should not be interpreted as a formal attributable burden estimate, as absolute numbers depend on local baseline utilization rates, population age structure, healthcare access patterns, case mix, and heat wave duration.

In this context, the potentially protective role of publicly accessible urban green spaces assumes particular importance. Recent evidence [84] demonstrates positive associations between urban green spaces and both objectively measured physical activity and mental health outcomes, with potentially beneficial effects on overall population well-being. However, accessibility is not sufficient: elements such as maintenance, proximity to residential areas, planning of interactive activities, and aspects related to perceived safety constitute fundamental determinants for maximizing benefits for physical and mental health [6]. These considerations are even more relevant in the context of heat waves, where green spaces may serve not only as areas of thermal mitigation but also as environments that foster community resilience [85].

A distinctive aspect of the present analysis, compared with the existing literature, lies in the shift in perspective regarding the outcomes of interest. While most studies have traditionally focused on direct clinical outcomes such as mortality and morbidity [86,87], the present work emphasizes healthcare service utilization as an indicator of heat wave impact. This approach is particularly relevant in the current context, where prevention strategies implemented in recent years—such as heat-health action plans and early warning systems—have proven effective in reducing the most severe clinical outcomes.

However, despite the success of preventive measures in mitigating mortality, pressure on healthcare services persists and represents a more sensitive and earlier indicator of the true impact of extreme heat exposures on the population [88]. This evidence is crucial for effective health planning in a scenario characterized by an increasing frequency and intensity of extreme climate events [89].

### 4.3. Implications for Policies and Public Health Practices

The findings of the present analysis provide important insights for informing health policies and public health practices in response to the effects of heat waves. A distinctive feature of this analysis lies in the assessment of not only acute healthcare utilization, but also access to outpatient services, highlighting a potential “chronic” impact of extreme heat exposures. This evidence underscores the need to move beyond traditional hospital preparedness plans, promoting stronger and genuine integration with community-based services and systematic implementation of weather-health alert systems [90,91], in line with the recommendations of the World Health Organization’s Heat-Health Action Plans [92].

These findings call for substantial rethinking of healthcare delivery in light of emergencies, challenges and evolving health needs. Population frailty, particularly among older adults, represents a multidimensional phenomenon that extends beyond clinical aspects to include social determinants such as isolation and loneliness [93]. Accordingly, it becomes necessary to move beyond the mere reorganization of the existing healthcare system and adopt a holistic, person-centered approach that prioritizes the life-course perspective and enables genuine healthy aging [80]. This paradigm requires the restructuring of care systems toward integrated health and social care models capable of addressing the complexity of vulnerable populations’ needs [6].

Particular attention should be given to the development and implementation of targeted risk communication strategies aimed at effectively informing vulnerable populations about preventive measures and appropriate behaviors during heat waves [94,95]. Risk communication represents a crucial element of prevention strategies and requires tailored approaches to effectively reach and engage high-risk groups.

Finally, in line with the One Health approach, it is imperative to consider the interconnectedness of human, animal, and environmental health, recognizing that the challenges posed by climate change demand intersectoral and multidisciplinary responses [96]. This approach calls for the coordinated involvement of health, environmental, urban planning, and social actors within an integrated public health governance framework capable of addressing the complexity and interdependence of health determinants in the context of climate change.

### 4.4. Strengths and Limitations

This systematic review and meta-analysis has several strengths. To our knowledge, it is the first study to comprehensively synthesize the evidence on the association between heat waves—conceptualized as multi-day extreme heat events—and healthcare utilization outcomes among frail older adults. By focusing on hospitalizations, emergency department visits, and outpatient care, this review extends beyond traditional mortality-based assessments and provides insights into the functional burden that heat waves place on healthcare systems. The review was conducted following the PRISMA guidelines, with a registered protocol, a comprehensive search strategy across multiple databases, and duplicate screening and data extraction. The use of random-effects meta-analytic models, prespecified sensitivity analyses, and risk of bias assessment tools tailored to different study designs further strengthens the robustness and transparency of the findings.

However, several limitations should be acknowledged. First, substantial heterogeneity was observed across studies, reflecting differences in heat wave definitions, exposure metrics, lag structures, outcome ascertainment, and analytical approaches. Although this heterogeneity limits direct comparability and precludes some subgroup analyses, the consistency in the direction of the associations across most studies supports the overall validity of the findings. Second, the operationalization of “frailty” and older age varied widely, with many studies relying on age-based thresholds rather than standardized frailty measures, potentially leading to exposure misclassification or dilution of effects. Third, evidence was unevenly distributed geographically, with a predominance of studies from high-income countries and a lack of data from low- and middle-income regions, where vulnerability to heat-related health impacts may be greater.

This geographical imbalance also points to a broader mismatch between scientific production and climate-related health vulnerability. In particular, regions in the Southern Hemisphere, including large areas of Africa and South America, were not represented in the available evidence, despite being highly exposed to climate-related hazards and often characterized by rapid demographic transition, increasing urbanization, and constrained healthcare resources. This gap is particularly relevant for older and frail populations, for whom the combined effects of population aging, social vulnerability, limited access to cooling resources, and reduced healthcare system resilience may amplify the impact of heat waves. Therefore, the lack of evidence from these settings may limit the global generalizability of the findings and highlights an important equity issue in climate-health research.

Additional limitations of the review process should also be acknowledged: Only studies published in English or Italian were included. Moreover, although reporting bias was explored through funnel plots and Egger’s tests, selective publication bias cannot be ruled out.

Finally, the observational nature of the included studies limits causal inference, and residual confounding—particularly related to socioeconomic factors, housing conditions, and access to cooling resources—cannot be excluded.

### 4.5. Implications for Future Research

Future research should aim to address several key gaps identified by this review. First, greater harmonization of heat wave definitions is needed to improve comparability across studies and facilitate pooled analyses, particularly with respect to threshold selection, duration criteria, and intensity metrics. Second, there is a need for studies that explicitly incorporate standardized measures of frailty, multimorbidity, and functional status, rather than relying solely on chronological age, to better characterize vulnerable subpopulations. Third, evidence on outpatient care and community-based healthcare utilization remains limited and warrants further investigation, as these settings may capture earlier or less severe heat-related health effects. Expanding research in underrepresented regions, particularly low- and middle-income countries, is also critical to ensure global relevance and equity in climate-health evidence. Future studies should prioritize settings where rapid population aging, urban growth, socioeconomic vulnerability, and limited healthcare system capacity may interact with increasing heat wave exposure, thereby generating a disproportionate burden among older and frail adults. Moreover, future studies should explore the interaction between heat waves and health system characteristics [97], including preparedness measures and adaptive capacity, to inform the development of climate-resilient healthcare systems and targeted public health interventions.

## 5. Conclusions

This analysis demonstrates that heat waves significantly impact healthcare service utilization beyond acute care, extending to outpatient services. Although relative risk increases appear modest, their population-level effects translate into a substantial public health burden, particularly in the context of demographic aging, increasing urbanization, and constrained healthcare system capacity.

A key contribution of this study is the identification of healthcare service utilization as a more sensitive indicator of heat wave impact compared with traditional clinical outcomes. Despite the effective prevention strategies in reducing severe outcomes, persistent healthcare pressure on healthcare services highlights ongoing challenges that require proactive planning.

These findings underscore the multidimensional nature of vulnerability and the need for holistic, life-course approaches that integrate health and social care. Policy implications include the implementation of comprehensive heat-health action strategies that combine hospital preparedness, community-based interventions, early warning systems, and targeted risk communication.

## Figures and Tables

**Table 1:** Characteristics of the included studies.

Author (Year)	Study Period	Study Design	Country	Level of Data Analysis	Number of Participants ≥ 60	Hospital Admission ≥ 60	Age of Participants (Years)	% Female	Population Baseline Characteristics
Achebak H. (2024) [65]	2006–2019	Observational ecological TS	Spain	National	NA	65–74 y: 1,495,388; 75–84 y: 2,124,135; >84 y: 1,418,394	All ages (older groups: 65–74; 75–84; ≥85)	54.8%	Population stratified by housing and municipality characteristics
Aghababaeian H. (2025) [74]	2013–2019	Observational ecological TS	Iran	Hospital/Health facility	NA	NA	All ages (older groups: 65–74; ≥75)	NA	Hospitalized patients with CV disease
Alho A. M. (2024) [75]	2000–2018	Retrospective observational ecological TS	Portugal	National	NA	NA	All ages (older group: ≥65)	NA	Resident population across 278 countries
Benmarhnia T. (2019) [25]	2006–2010	Quasi-experimental observational	USA	City	NA	158	≥65	NA	Medicare fee-for-service beneficiaries
Bobb J. F. (2014) [26]	1999–2010	Observational matched TS	USA	National	23,700,000	NA	≥65	NA	Medicare fee-for-service beneficiaries in 1943 counties
Borg M. (2019) [40]	2003–2014	Observational ecological TS	Australia	City	NA	NA	All ages (older group: ≥65)	NA	Hospitalized patients with KD
Bujosa Mateu A. (2024) [66]	2005–2021	Observational ecological TS	Spain	Hospital/Health facility	NA	107,053	All ages (older groups: 65–80; >80)	51.2%	Patients admitted to ICU or ED
Bundo M. (2021) [72]	1973–2017	Observational ecological TS	Switzerland	Hospital/Health facility	NA	13,596	All ages (older group: ≥65)	50.1%	Hospitalized patients with MD
Bustinza R. (2013) [69]	2005–2010	Observational ecological descriptive	Canada	Regional	NA	NA	All ages (older groups: 65–74; ≥75)	NA	ED visits for all causes
Campbell S. L. (2019) [51]	2008–2016	Observational time-stratified CS	Australia	Hospital/Health facility	NA	160,500	All ages (older group: ≥65)	48.3%	ED presentations for all causes
Chu L. (2022) [60]	2003–2015	Observational CS	Vietnam	Provincial	NA	Glomerular diseases: 233; renal tubulo-interstitial disease: 380; chronic kidney disease: 8254; urolithiasis: 5538	All ages (older groups: 60–99)	NA	Hospitalized patients with KD
Dang T. N. (2019) [61]	2010–2013	Observational ecological TS	Vietnam	National	NA	217,000	All ages (older group: ≥65)	51.8%	Hospitalized patients for all causes
Deng S. Z. (2023) [52]	2016–2021	Observational ecological TS	China	City	NA	94,244	All ages (older group: ≥60)	44.6%	Hospital admissions for asthma
Fuhrmann C. M. (2016) [27]	2007–2011	Observational ecological event-based TS/case series	USA	National	NA	NA	All ages (older group: ≥65)	NA	Patients admitted to ED
Gossack-Keenan K. (2024) [70]	2021	Retrospective descriptive observational	Canada	Regional	NA	139	≥16 (median age: 84.4)	54.6%	Adults hospitalized for heatstroke
Gronlund C. J. (2014) [28]	1992–2006	Observational time-stratified CS	USA	National	NA	NA	≥65	NA	Medicare beneficiaries residing in 114 US cities
Ha S. (2014) [29]	1994–2000	Observational time-stratified CS	USA	Regional	NA	12,195	≥65	58.6%	Adults hospitalized for first-ever stroke
Hansen A. (2008) [41]	1993–2006	Observational ecological TS	Australia	City	NA	NA	All ages (older groups: 65–74; ≥75)	NA	Metropolitan residents hospitalized with primary diagnoses of MD, CV disease or BD
Hansen A. (2008)2 [42]	1995–2006	Observational ecological TS	Australia	City	NA	65–84 y: 1137; >85 y: 213	All ages (older groups: ≥65; ≥85)	NA	Metropolitan residents hospitalized with primary diagnosis of KD
Hopp S. (2018) [30]	1999–2010	Observational matched ecological TS	USA	National	23,700,000	NA	≥65	NA	Medicare fee-for-service beneficiaries
Huang Y. S. (2023) [53]	2014–2019	Observational time-stratified CS	China	Multi	NA	28,045	All ages (older group: ≥65)	42.1%	Hospitalized patients with KD in 23 Chinese sites
Jung Y. S. (2025) [31]	2000–2016	Observational time-stratified CS	USA	Regional	NA	27,827	≥65	62%	Medicare fee-for-service beneficiaries
Kim H. (2024) [32]	2016–2019	Observational retrospective ecological TS	USA	National	5,448,499	NA	≥65	66%	Dually eligible Medicare–Medicaid beneficiaries
Knowlton K. (2009) [33]	2006	Observational ecological descriptive	USA	Regional	NA	ED visits: 87,932; hospitalizations: 56,230	All ages (older group: ≥65)	NA	Residents with ED visits or EA
Lai E. T. (2025) [54]	2012–2018	Observational time-stratified CS	China	City	NA	CV admissions: 112,931; respiratory admissions: 140,744	≥65	NA	Adults admitted via ED for CV or respiratory disease
Lindstrom S. J. (2013) [43]	2009	Observational ecological event-based descriptive	Australia	Hospital/health facility	Study week: ED 271, total hospital admissions 418, GM admissions 78; control period: ED 555, total hospital admissions 1015, GM admissions 116	NA	≥70	52%	Patients with ED visits or EA
Liss A. (2017) [34]	1991–2006	Observational ecological TS	USA	City	NA	701	≥65	NA	Medicare beneficiaries in metropolitan areas
Liss A. (2019) [35]	1991–2006	Observational ecological TS	USA	Regional	31,633,400	40,019	≥65	NA	Medicare beneficiaries residing in the USA
Liu X. (2019) [55]	2010	Observational symmetric bidirectional CS	China	City	NA	NA	≥65	NA	Hospital visits for mental disorders among residents
Martínez-Solanas È. (2019) [67]	1997–2013	Observational ecological TS	Spain	Provincial	NA	CV admissions: 65–74 y 1,106,467, 75–84 y 1,498,936, ≥85 716,250; cerebrovascular admissions: 65–74 y 330,284, 75–84 y 471,232, ≥85 219,521; respiratory admissions: 65–74 y 881,821, 75–84 y 1,307,356, ≥85 742,499	All ages (older groups: 65–74; 75–84; ≥85)	NA	Residents with non-scheduled EA recorded in the CMBD registry
Mastrangelo G. (2007) [76]	2002–2003	Observational ecological TS	Italy	Regional	NA	NA	≥75	NA	Residents of the Veneto region
Mayner L. (2010) [44]	2009	Observational ecological descriptive event-based	Australia	Hospital/health facility	NA	HW1: 512 ED visits	≥65	NA	ED presentations among community-dwelling adults
McTavish R. K. (2018) [71]	2005–2012	Observational population-based matched case–control	Canada	Provincial	Acute Kidney Injury (AKI) cases: 52,913; controls: 174,222	NA	≥65	49%	Residents with at least one year of baseline medication data
Nhung N. T. T. (2023) [64]	2010–2017	Observational ecological TS	Vietnam	Provincial	NA	Respiratory diseases: Ninh Thuan 543, Ca Mau 551; CV diseases: 1338	All ages (older group: ≥60)	NA	Patients admitted to one hospital per province
Nitschke M. (2011) [45]	1993–2009	Observational population-level case series (ecological)	Australia	City	NA	NA	All ages (older groups: 65–74; ≥75)	NA	Metropolitan resident population
Ogbomo A. S. (2017) [24]	2000–2009	Observational time-stratified CS	USA	Hospital/health facility	NA	CV diseases: 151,842; respiratory diseases: 54,934; Diabetes mellitus: 7207; renal diseases: 10,788; acute myocardial infarction: 16,223; all natural causes: 453,687	All ages (older group: ≥65)	NA	Acute-care inpatient admissions among residents
Parry M. (2019) [46]	2001–2013	Observational time-stratified CS	Australia	Hospital/health facility	NA	Ischemic heart disease: 39,837; heart failure: 21,251; cardiac arrest: 1059; heart arrhythmia: 20,221; conduction disorders: 2429; hypertensive diseases: 2288	All ages (older group: ≥65)	NA	EA in hospitals
Phung D. (2016) [62]	2004–2013	Observational ecological TS	Vietnam	City	NA	NA	All ages (older group: 65–84; ≥85)	NA	Patients admitted to the two largest hospitals
Ponjoan A. (2017) [68]	2006–2013	Observational population-based self-controlled case series	Spain	Hospital/health facility	NA	11,682	All ages (older group: ≥65)	45%	Adults residing in the community hospitalized for CV disease
Ragettli M. S. (2019) [73]	2015	Observational ecological event-based	Switzerland	National	NA	Jun–Aug 2015: 65–74 y 17,455, ≥75 35,669; July 2015 65–74 y 6074, ≥75: 12,580	All ages (older groups: 65–74; ≥75)	NA	Swiss resident population
Santodomingo M. (2024) [36]	2012–2019	Observational time-stratified CS	USA	Hospital/health facility	NA	8,744,001	≥65	57.1%	Adults presenting to 324 non-federal hospital EDs
Schaffer A. (2012) [47]	2006–2011	Observational ecological TS	Australia	Regional	NA	NA	All ages (older group: ≥75)	NA	Resident population in HW-affected areas
Semenza J.C. (1999) [37]	1994–1995	Observational ecological descriptive	USA	City	NA	3230	All ages (older group: ≥65)	NA	Urban residents served by 47 acute-care hospitals
Smith S. (2016) [77]	2012–2014	Observational ecological descriptive	UK	National	NA	NA	All ages (older groups: 65–74; ≥75)	NA	GP covered by national real-time syndromic surveillance
Sohail H. (2020) [78]	2001–2017	Observational ecological TS	Finland	Regional	NA	NA	All ages (older groups: 65–74; ≥75)	NA	Residents with non-elective hospital admissions for CV or respiratory disease
Toloo G.S. (2014) [48]	2000–2012	Observational ecological TS	Australia	City	NA	NA	All ages (older groups: 65–74; ≥75)	NA	Adults presenting to 11 hospital EDs
Tong S. (2014) [49]	1996–2005	Observational ecological TS	Australia	City	NA	NA	All ages (older groups: 65–74; ≥75)	64.9%	Urban population of a subtropical city
Trang P.M.(2016) [63]	2008–2012	Observational ecological TS	Vietnam	City	NA	1595	All ages (older group: ≥60)	23.9%	Patients with emergency psychiatric hospital admissions
Wang Y. (2016) [38]	1999–2010	Observational matched ecological TS	USA	Regional	23,500,000	NA	≥65	NA	Medicare fee-for-service beneficiaries in 1916 counties
Wilson L.A. (2013) [50]	1997–2007	Observational time-stratified CS	Australia	City	NA	NA	≥65	NA	Residents living in the metropolitan region
Yin J. (2025) [56]	2018–2019	Observational ecological TS	China	Provincial	NA	262,400	All ages (older group: ≥60)	42%	Hospitalized patients with stroke in 122 districts
Yong K.H. (2023) [57]	2010–2019	Observational ecological TS	China	City	NA	2925	≥60 (age groups: 60–69, 70–79, ≥80)	45%	Adults hospitalized for all causes
Zhang A. (2019) [59]	2010–2012	Observational time-stratified CS	China	City	NA	1828	All ages (older group: ≥65)	39%	Outpatients attending a county hospital
Zhang H. (2024) [58]	2010–2014	Observational ecological TS	China	City	NA	NA	All ages (older group: ≥60)	50%	Outpatient visits for mental disorders among urban residents
Zhang K. (2015) [39]	2007–2011	Observational ecological TS	USA	City	NA	NA	All ages (older group: ≥65)	NA	GP

BD = behavioral disorder; CMBD = Conjunto Mínimo Básico de Datos; CS = case-crossover; CV = cardiovascular; EA = emergency admission; ED = emergency department; GM = general medical; GP = general population; ICU = intensive care unit; KD = kidney disease; MD = mental disorder; TS = time series; UK = United Kingdom; USA = United States of America; y = year.

**Table 2:** Characteristics of heat wave exposure assessment across included studies.

Author (Year)	Method Used to Measure Exposure	HW Definition	HW Intensity Index	Season of Measurement	Years/Months of Measurement	Lag Structure (Days)	Baseline Reference Period	Type of Comparison
Achebak H. (2024) [65]	Tmax, Tmin and Tmean at 2 m from E-OBS gridded dataset	≥2 or ≥4 consecutive days with Tmean > 95 P	NA	Summer/warm season	2006–2019/June September	0–7 DLNM	NA	HW days vs. non-HW days
Aghababaeian H. (2025) [74]	Daily meteorological data from City Meteorological Center and Department of Environment	Definition 1: >2 days with Tmean ≥ 95 P; Definition 2: >3 days with Tmax ≥ 90 P	NA	Summer/warm season	2013–2019	0, 0–2, 0–6, and 0–13	NA	HW days vs. non-HW days
Alho A. M. (2024) [75]	Daily temperature from E-OBS gridded data; EHF based on 90 P (1961–1990)	3 consecutive days with Tmean > 90 P	EHF significance based on 3-day deviation of Tmean above 90 P (reference period 1961–1990)	Summer/warm season	2000–2018/May–September	NA	1961–1990	HW days vs. non-HW days
Benmarhnia T. (2019) [25]	Daily temperature from LaGuardia Airport weather station from NOAA; PM2.5 and O_3_ from EPA AQS	≥2 days with HI > 95 °F or ≥1 day with HI > 100 °F	HI intensity thresholds: ≥100 °F for ≥1 day or ≥95 °F for ≥2 consecutive days	Summer/warm season	2006–2010/May–September	NA	2004–2010	HW days vs. non-HW days
Bobb J. F. (2014) [26]	Daily meteorological data from NCDC	2 or ≥4 consecutive days with Tmean > 97 P, >98 P or >99 P	NA	All seasons	1999–2010/January–December	0–7	NA	HW days vs. non-HW days
Borg M. (2019) [40]	Daily meteorological data from Australian BoM	Definition 1: ≥5 days with Tmax ≥ 35 °C and/or ≥3 days with Tmean ≥ 40 °C; Definition 2: ≥3 days with Tmax > 95 P	NA	Summer/warm season	2003–2014/July–March	0–10	1997–2014	HW days vs. non-HW days
Bujosa Mateu A. (2024) [66]	Daily meteorological data from Spanish Meteorological Agency OpenData system	≥2 consecutive days with Tmax ≥ 35 °C	NA	All seasons	2005–2021/January–December	0–7 DLNM	NA	HW days vs. non-HW days
Bundo M. (2021) [72]	Tmean from MeteoSwiss	≥2 or ≥3 consecutive days with temperature ≥90 P or ≥95 P	NA	Summer/warm season	1973–2017/May–September	0–3	1973–1989 (May–September)	HW days vs. non-HW days
Bustinza R. (2013) [69]	Daily temperature from regional reference stations (Environment Canada)	3 consecutive days with Tmin and Tmax exceeding predefined heat-risk thresholds	NA	Summer/warm season	2010/July	NA	2005–2009 (July)	(1) Comparison period corresponded to the same days of the week during the years 2005, 2006, 2007, 2008, and 2009; (2) corresponded to the dates closest to the 2010 impact period
Campbell S. L. (2019) [51]	Daily temperature from BoM and Tasmania EPA air quality monitoring network, BLANkET	2 consecutive days with Tmax and Tmin exceeding reference-period thresholds; heat waves identified using the EHF	NA	All seasons	2008–2016/January–December	NA	2008–2016 (January–December)	HW days vs. non-HW days
Chu L. (2022) [60]	Daily temperature from provincial hydrometeorological stations or nearest airport stations	EH days defined as Tmax > 95 P	NA	Summer/warm season	2003–2015/southern provinces: January–December; northern provinces: May–October	Single (0–14) and cumulative exposures (0–6)	2003–2015	HW days vs. non-HW days
Dang T. N. (2019) [61]	Daily temperature from Tan Son Nhat Airport or NOAA NCDC	2 consecutive days with Tmean > 97 P	NA	All seasons	2010–2013	0–1,0–3, and 0–7	1 January 2010–31 October 2013	HW days vs. non-HW days
Deng S. Z. (2023) [52]	Daily temperature, humidity and atmospheric pressure from National Meteorological Administration	≥2 consecutive days with Tmean > 90 P (30.0 °C)	NA	Summer/warm season	2016–2021/May–October	0–3	Non-HW days during the warm season of the study period	HW days vs. non-HW days
Fuhrmann C. M. (2016) [27]	Event-based exposure defined using NWS heat advisories and warnings	≥5 consecutive days with Tmax > 110 °F	HI-based intensity categories embedded in NWS criteria (Heat Advisory, Excessive Heat Watch/Warning)	Summer/warm season	2007–2011/June–August	NA	Control periods defined as four non-HW windows per event, averaged as baseline	Observed vs. expected ED visits
Gossack-Keenan K. (2024) [70]	Daily temperature from Vancouver International Airport compared with 30-year baseline	≥3 consecutive days with Tmax and Tmin > 90 P	NA	Summer/warm season	2021/June	NA	1991–2020 30-year climatological baseline used for P thresholds	No non-HW control period; analyses are within heatstroke cohort (survivors vs. non-survivors)
Gronlund C. J. (2014) [28]	Tmean and dew point from NCDC station data	≥2 consecutive days with Tmean > 95 P	Threshold exceedance of 2-day mean apparent temperature > 95 P	Summer/warm season	1992–2006/May–September	0–1, 2–3, 4–5, 6–7	75 P of apparent temperature used as reference for moderate vs. EH contrasts	HW days vs. non-HW days
Ha S. (2014) [29]	Tmean and relative humidity from NCDC (Pittsburgh International Airport)	≥2 consecutive days with Tmean > 95 P	HW intensity assessed as total number of HW days within the preceding 4-day window	Summer/warm season	1994–2000/May–September	0–3	Non-HW days during the warm season	HW days vs. non-HW days
Hansen A. (2008) [41]	Tmax from Australian BoM	≥3 consecutive days with Tmax ≥ 35 °C	NA	Summer/warm season	1993–2006/October–March	NA	Non-HW days during the warm season	HW days vs. non-HW days
Hansen A. (2008) 2 [42]	Tmax and Tmin from Australian BoM	≥3 consecutive days with Tmax ≥ 35 °C (95 P)	NA	Summer/warm season	1995–2006/November–March	NA	Non-HW days during the warm season	HW days vs. non-HW days
Hopp S. (2018) [30]	Tmean from gridded meteorological data from NCEI	≥2 or ≥4 consecutive days with Tmean > 97 P, >98 P or >99 P	NA	All seasons	1999–2010/January–December	NA	Non-HW days matched on county and calendar time	HW days vs. non-HW days
Huang Y. S. (2023) [53]	Daily temperature from China Meteorological Administration	≥2, ≥3 or ≥4 consecutive days with Tmean > 90 P, >95 P or >97.5 P	NA	Summer/warm season	2014–2019/May–September	0–7 DLNM	Matched non-HW days by day of week, month, and year	HW days vs. non-HW days
Jung Y. S. (2025) [31]	HI calculated from Tmax and dew point using PRISM data	≥2 consecutive days with HI ≥ 90 P	NA	Summer/warm season	2000–2016/May–September	NA	Non-HW days within the same month, year, and day of week	HW days vs. non-HW days
Kim H. (2024) [32]	Tmax and relative humidity from Daymet (ORNL DAAC)	≥3 consecutive days with Tmax ≥ 90 P or ≥97 P	NA	Summer/warm season	2016–2019/May–September	NA	Non-HW days within the same ZCTA and calendar period	HW days vs. non-HW days
Knowlton K. (2009) [33]	Event-based exposure defined using meteorological observations	NA	NA	Summer/warm season	2006/July–August	NA	Pre- and post-HW comparison periods	HW days vs. non-HW days
Lai E. T. (2025) [54]	Tmax and Tmin from Hong Kong Observatory	≥3 or ≥5 consecutive days with Tmax ≥ 33 °C or Tmin ≥ 28 °C	NA	Summer/warm season	2012–2018/June–September	0–21	Non-HW days within the same month and year (time-stratified case-crossover)	HW days vs. non-HW days
Lindstrom S. J. (2013) [43]	Tmax and Tmin from Australian BoM	≥3 consecutive days with Tmax > 43 °C or ≥6 consecutive days with Tmin > 20 °C	NA	Summer/warm season	2009/January–February	NA	Historical control period including the week preceding HW and matched calendar weeks in 2007–2008	Pre–post comparison and historical matched-week comparison within the same hospital
Liss A. (2017) [34]	Tmax and Tmin from NOAA Global Summary of the Day	≥2 consecutive days with Tmean > 20.8 °C and Tmin > 18.6 °C	NA	Summer/warm season	1991–2006/May–September	NA	Non-HW days within the same calendar years	HW days vs. non-HW days
Liss A. (2019) [35]	Nationwide daily meteorological observations	≥2 consecutive days with Tmin > 90 P	NA	Summer/warm season	1991–2006/June–September	NA	Non-HW days within the same climate region and calendar year	HW days vs. non-HW days
Liu X. (2019) [55]	Tmax from Jinan BoM	≥3 consecutive days with Tmax ≥ 35 °C	NA	Summer/warm season	2010/June–August	0–5	Non-HW days within the same summer period	HW days vs. non-HW days
Martínez-Solanas È. (2019) [67]	Tmax from ECA&D dataset for provincial capitals	≥4 consecutive days with temperature > 95 P	NA	Summer/warm season	1997–2013/June–September	0–21 DLNM	Temperature P corresponding to minimum hospitalizations	Before–after comparison (1997–2002 vs. 2004–2013)
Mastrangelo G. (2007) [76]	Tmax and humidity from meteorological stations; Humidex calculated and regionally averaged	≥3 consecutive days with Humidex ≥ 40 °C	Humidex as continuous intensity metric during HW days	Summer/warm season	2002–2003/June–August	NA	Days outside HW periods within the same summer	HW days vs. non-HW days
Mayner L. (2010) [44]	Tmax and Tmin from Australian BoM	≥5 consecutive days with Tmax ≥ 35 °C or ≥3 consecutive days with Tmax ≥ 40 °C	NA	Summer/warm season	2009/January–February and November	NA	Two weeks before and two weeks after each HW	Pre-HW vs. during HW vs. post-HW comparisons within the same hospital
McTavish R. K. (2018) [71]	Daily temperature from ICES Global Environmental Multiscale Surface database	≥3 consecutive days with Tmax ≥ 95 P	NA	Summer/warm season	2005–2012/April–September	0–2	All days not meeting the HW definition	HW days vs. non-HW days
Nhung N. T. T. (2023) [64]	Daily temperature from provincial hydrometeorological stations	≥3 consecutive days with Tmax > 90 P	NA	All seasons	2010–2017/January–December	0–4	Non-HW days during the study period	HW days vs. non-HW days
Nitschke M. (2011) [45]	Tmax from South Australia BoM	≥3 consecutive days with Tmax ≥ 35 °C	NA	Summer/warm season	1993–2009/Oct–Mar	NA	Non-HW days within the warm season	HW days vs. non-HW days
Ogbomo A. S. (2017) [24]	Tmean from NCDC stations	≥1 day of EH or ≥2, ≥3 or ≥4 consecutive days with Tmean > 95 P, >97 P or >99 P	P-based intensity defined by P thresholds (95/97/99) and duration (1–4 consecutive days)	Summer/warm season	2000–2009/May–September	0–1	Non-EH/non-HW control days matched by month and day of week	HW days vs. non-HW days
Parry M. (2019) [46]	Daily meteorological data from Australian BoM	≥2 consecutive days with Tmax, Tmean and Tmin ≥ 90 P	NA	Summer/warm season	2001–2013/November–March	0–2	Referent days matched by month and day of week	HW days vs. non-HW days
Phung D. (2016) [62]	Daily meteorological data from Southern Regional Hydro-Meteorological Centre	≥2 consecutive days with Tmean > 99 P	P-based intensity using P thresholds (95/97/99) with fixed duration ≥2 consecutive days	All seasons	2004–2013/February–December	0–7	Non-HW days during the study period	HW days vs. non-HW days
Ponjoan A. (2017) [68]	Hourly temperature from automatic weather stations	≥3 consecutive days with Tmax > 95 P	NA	Summer/warm season	2006–2013/July–August	NA	Non-HW days within July–August observation periods	HW days vs. non-HW days
Ragettli M. S. (2019) [73]	NA	NA	NA	Summer/warm season	2015/June–August	NA	Expected EA estimated from stratum-specific models (2012–2014)	Observed EHA June–Aug 2015 vs. expected EHA for the same period predicted from 2012 to 2014
Santodomingo M. (2024) [36]	Daily temperature from PRISM gridded dataset at ZIP code level	2 consecutive days above P-based thresholds	P-based intensity categories using P thresholds (95/97.5/99) and duration classes (HWD1–HWD6)	Summer/warm season	2012–2019/May–September	NA	Non-EH control days matched by month and day of week	HW days vs. non-HW days
Schaffer A. (2012) [47]	Tmax and night-time Tmin from Australian BoM	NA	NA	Summer/warm season	2011/January–February	0–4	Non-HW days during summers 2006/07–2010/11	HW days vs. non-HW days
Semenza J.C. (1999) [37]	Daily maximum HI from O’Hare Airport	NA	HI as apparent temperature intensity metric (maximum HI recorded = 119 °F)	Summer/warm season	1995/July	0–2	Same calendar weeks in 1994 and the immediately preceding week in 1995 (non-HW)	HW days vs. non-HW days
Smith S. (2016) [77]	Daily CET from UK Met Office Hadley Centre	Heat-Health Watch Warning System, level 3	NA	Summer/warm season	2012–2014/June–September	NA	Same calendar weeks in non-HW summers (2012 and 2014) without level 3 heat alerts	HW days vs. non-HW days
Sohail H. (2020) [78]	Tmean from Finnish Meteorological Institute	≥4 consecutive days with temperature > 90 P or ≥3 consecutive days >95 P	P-based Tmean intensity using 90 and 95 P	Summer/warm season	2001–2017/June–August	0–1	May–August 2001–2017	HW days vs. non-HW days
Toloo G.S. (2014) [48]	City-averaged daily Tmax from Australian BoM	≥2 consecutive days with Tmax ≥ 34 °C (95 P) or ≥37 °C (99 P)	P-based Tmax intensity using 90/95/99 P thresholds	Summer/warm season	2000–2012	0–3	Historical warm-season temperature P from 1996 to 2005	HW days vs. non-HW days
Tong S. (2014) [49]	Tmax, Tmin and Tmean from meteorological stations	≥2 consecutive days with Tmean ≥ 90 P, ≥95 P or ≥98 P	P-based intensity tiers	Summer/warm season	1996–2005	0–3	Historical warm-season temperature P from the study period	HW days vs. non-HW days
Trang P.M.(2016) [63]	Tmax and Tmean from official local monitoring stations	≥3 or ≥7 consecutive days with Tmean ≥ 90 P (35 °C)	P-based Tmax intensity using 90/95/99 P thresholds	Summer/warm season	2008–2012	0–7 DLNM	Local climatological distribution of daily Tmax during warm months (May–September)	HW days vs. non-HW days
Wang Y. (2016) [38]	Tmean from NCDC stations	≥2 consecutive days with Tmean > 97 P	HW severity categorized using EHF thresholds (extreme >80 P; severe EHF >1); alternative intensity proxies using 97/98/99 P moving averages	All seasons	1999–2010	0–3	County-specific historical daily Tmean distribution (1999–2010) used to define local P thresholds	HW days vs. non-HW days
Wilson L.A. (2013) [50]	Tmax and maximum apparent temperature from Australian BoM	Definition 1: ≥3 days with Tmean > 99 P; Definition 2: ≥2 days with Tmax > 95 P	Combined intensity definition using 95/99 P thresholds and EHF severity (EHF > 1; >80 P)	Summer/warm season	1997–2010/September–February	0–3	Monthly temperature distributions by Sydney GMR climate zone (1997–2010)	HW days vs. non-HW days
Yin J. (2025) [56]	Tmax, Tmin, Tmean, relative humidity and pressure from China Meteorological Data Network	≥3 consecutive days with Tmax ≥ 35 °C	NA	Summer/warm season	2018–2019/May–September	0–10	NA	HW days vs. non-HW days
Yong K.H. (2023) [57]	Daily apparent temperature from meteorological stations	≥3 consecutive days with Tmean ≥ 37 °C (97.5 P)	NA	Summer/warm season	2010–2019/May–September	0–7 DLNM	NA	HW days vs. non-HW days
Zhang A. (2019) [59]	Tmax and Tmean	≥3 consecutive days with Tmax > 35 °C	NA	Summer/warm season	2010–2012/July–September	0–7 DLNM	NA	HW days vs. non-HW days
Zhang H. (2024) [58]	Tmean, relative humidity and pressure from Guangzhou BoM	≥2, ≥3 or ≥4 consecutive days with Tmean > 90 P, >92.5 P or >95 P	HW intensity categorized as low (HW1–3), moderate (HW4–6), high (HW7–9)	Summer/warm season	2010–2014/May–October	0–10	May–October 2010–2014 (used to define percentiles)	HW days vs. non-HW days
Zhang K. (2015) [39]	Daily temperature from NWS	≥2 consecutive days with Tmean ≥ 95 P, ≥97 P or ≥99 P	NA	Summer/warm season	2007–2011	0–7 DLNM	NA	HW days vs. non-HW days

AQS = Air Quality System; BLANkET = Base Line Air Network of EPA Tasmania; BoM = Bureau of Meteorology; CET = Central England Temperature; DLNM = distributed lag non-linear model; ECA&D = European Climate Assessment & Dataset; EH = extreme heat; EHF = Excess Heat Factor; EPA = Environmental Protection Agency; E-OBS = European Observations; HI = Heat Index; ICES = Institute for Clinical Evaluative Sciences; NCDC = National Climatic Data Center; NCEI = National Centers for Environmental Information; NOAA = National Oceanic and Atmospheric Administration; NWS = National Weather Service; ORNL DAAC = Oak Ridge National Laboratory Distributed Active Archive Center; P = percentile; PRISM = Parameter-elevation Relationships on Independent Slopes Model; Tmax = daily maximum temperature; Tmin = daily minimum temperature; Tmean = daily mean temperature; UK = United Kingdom.

**Table 3:** Measurement of health service utilization outcomes and analytical approaches in included studies regarding the relationship between heat waves (HW) and healthcare utilization in frail older adults (age ≥ 60 years).

Author (Year)	Method Used to Measure Healthcare Outcome	Unit of Analysis	Type of Outcome	Health Event	Specific Disease	Statistical Method Used to Analyze HW-Outcome Association	Main Results for Adults Aged ≥60 Years (with 95% CIs)	Effect Measure Type	Variables of Adjustment
Achebak H. (2024) [65]	Records of hospital admissions from the INE	Daily counts	ED visits	All-cause ED	NA	Quasi-Poisson GLM with DLNM	65–74 y: 1.05 (1.03; 1.08); 75–84 y: 1.10 (1.08; 1.13); >84 y: 1.19 (1.17; 1.22)	RR	Relative humidity, PM_2.5_, PM_10_, NO_2_, O3
Aghababaeian H. (2025) [74]	Hospital admission records from Dezful University of Medical Sciences using ICD-10 codes for cardiovascular diseases	Daily counts	Hospitalizations	CV diseases	NA	Quasi-Poisson GLM with DLNM	For age 65–74: HW1 (heat wave defined as average daily temperature): lag 0: 39.73 (−26.66, 166.23) lag 0–2: 54.52 (−77.43, 276.25) lag 0–6: −8.93 (−234.13, 341.89) lag 0–10: −25.88 (−373.22, 511.10) HW2 (heat wave defined as maximum daily temperature) lag 0: 12.36 (−6.47, 35.00) lag 0–2: 27.93 (−9.54, 71.06) lag 0–6: 33.86 (−35.00, 111.01) lag 0–10: 22.01 (−80.50, 136.41)	CER	Age, sex
Alho A. M. (2024) [75]	Hospital admission records from the Hospital Morbidity Database coded using ICD-9-CM and ICD-10	Daily counts	Hospitalizations	All-cause ED	NA	NB regression	1.17 (1.16–1.18)	IRR	Age, sex
Benmarhnia T. (2019) [25]	Medicare inpatient claims data coded using ICD-9-CM	Daily counts	Hospitalizations	All-cause ED	NA	LR using DID	0.80 (0.27–1.33)	DID: 2006–2007 vs. 2009–2010	Tmax, O_3_, PM_2.5_
Bobb J. F. (2014) [26]	Hospital discharge diagnosis codes grouped by primary ICD-9 diagnosis	Daily counts	Hospitalizations	All-cause ED	NA	Log-linear mixed-effects regression	1.18 (1.12–1.25) for fluid and electrolyte disorders, 1.14 (1.06–1.23) for renal failure, 1.10 (1.04–1.16) for urinary tract infections, 1.06 (1.00–1.11) for septicemia, and 2.54 (2.14–3.01) for heat stroke	RR	Age
Borg M. (2019) [40]	Hospital discharge records coded using ICD-10	Daily counts	ED visits	Renal diseases	NA	NB regression	EHF HW: ED 1.06 (1.01–1.11); inpatient 1.01 (0.95–1.07). EHF Severe: ED 1.20 (1.10–1.31); inpatient 1.06 (0.95–1.18)	IRR	Age, sex
Bujosa Mateu A. (2024) [66]	Hospital discharge minimum dataset	Daily counts	ED visits	All-cause ED	CV, neurological, infectious diseases	Quasi-Poisson GLM	66–80 y: 1.01 (0.91–1.11); >80 y: 1.09 (0.98–1.21)	RR	Age, sex, income, setting
Bundo M. (2021) [72]	Psychiatric hospital admission records from the University Psychiatric Hospital of Bern coded using ICD-10	Daily counts	Hospitalizations	Neurological diseases	NA	Quasi-Poisson GLM with DLNM	1.04 (0.97–1.11)	RR	Age, sex, relative humidity, rainfall, atmospheric pressure, PM_10_, NO_2_, O_3_
Bustinza R. (2013) [69]	Daily administrative reports from ED and hospitals from the Ministry of Health and Social Services	Daily counts	ED visits	All-cause ED	NA	Poisson-based crude rate estimation with log-normal approximation for CI	65–74 y: 2005–2009: 3.99 (3.70–4.31); 2010: 4.40 (3.77–5.14). >74 y: 2005–2009: 15.99 (15.34–16.66); 2010: 21.22 (19.64–22.93)	Mortality rate	NA
Campbell S. L. (2019) [51]	Public hospital admission records from the Tasmanian Health Service coded using ICD-10	Daily counts	ED visits	All-cause ED	Diabetes, respiratory, CV, neurological diseases	Conditional multivariate LR	1.06 (0.97–1.10)	OR	Age, gender, socioeconomic index for areas, diagnostic group
Chu L. (2022) [60]	Hospital discharge records coded using ICD-10	Individual events	Hospitalizations	Renal diseases	NA	Conditional LR with random-effects meta-analysis	Glomerular diseases: 1.06 (0.91–1.24); renal tubulo-interstitial disease: 1.05 (0.92–1.20); chronic kidney disease: 1.07 (1.01–1.15); urolithiasis: 1.07 (1.01–1.14)	OR	Relative humidity
Dang T. N. (2019) [61]	Hospital admission records from two hospitals (Gia Dinh People’s Hospital and People’s Hospital) coded using ICD-10	Daily counts	Hospitalizations	CV, respiratory diseases	NA	Quasi-Poisson time-series regression with DLNM	1.28 (1.14–3.45)	RR	Tmax, Tmin, Tmean, relative humidity, age, sex
Deng S. Z. (2023) [52]	Daily asthma-related ED and outpatient visits from the Shenzhen Health Development Research and Data Management Center coded using ICD-10	Daily counts	Outpatient care + ED visits	Respiratory diseases	Asthma	Time-series quasi-Poisson regression with DLM	Lag 3: 1.01 (0.98–1.04); cumulative effect Lag 0–3: 0.98 (0.90–1.07)	RR	Long-term trends and seasonality, day of the week, public holidays, relative humidity, atmospheric pressure, O_3_
Fuhrmann C. M. (2016) [27]	EDSSS data from the North Carolina Disease Event Tracking and Epidemiologic Collection Tool	Daily counts	ED visits	All-cause ED	CV, respiratory, endocrine, neurological, renal diseases	Observed vs. expected comparison with 95% CI	8.9 ED visits per 100,000	ED visit rate for HRI	NA
Gossack-Keenan K. (2024) [70]	Hospital medical records review	Individual patients	ED visits	Neurological diseases	Stroke, delirium	Descriptive analyses with Mann–Whitney U tests and LR for mortality	In-hospital mortality: 16/139 (11.5%)	Mortality percentage	NA
Gronlund C. J. (2014) [28]	Medicare inpatient billing records for emergency hospital admissions	Daily counts	Hospitalizations	All-cause ED	CV, renal, respiratory diseases	Time-stratified case-crossover with Poisson regression and multivariate meta-analysis	2-day HW: 1.01 (1.01, 1.02); 4-day HW: 1.03 (1.02, 1.04); 6-day HW: 1.03 (1.02, 1.04); 8-day HW: 1.02 (1.00, 1.03)	RR	Year/month/day-of-week, controlling seasonality and long-term trends
Ha S. (2014) [29]	Hospital discharge records from the Pennsylvania Health Care Cost Containment Council	Individual events	Hospitalizations	CV diseases	Stroke	Time-stratified case-crossover with conditional LR	Lag 0: 0.99 (0.88–1.11); Lag 1: 1.00 (0.89–1.13); Lag 2: 1.17 (1.04–1.31); Lag 3: 1.11 (0.99–1.24)	OR	Relative humidity, PM_10_, O_3_
Hansen A. (2008) [41]	Hospital discharge records from the ISAAC	Daily counts	Hospitalizations	Mental diseases	NA	Fixed-effects Poisson regression (NB when over-dispersed) with threshold analysis	65–74 y: Males 1.13 (0.95–1.36), Females 1.09 (0.94–1.26); ≥75 y: Males 1.17 (1.01–1.34), Females 1.19 (1.07–1.32)	IRR	Seasonality, long-term trends
Hansen A. (2008) 2 [42]	Hospital discharge records from the ISAAC	Daily counts	Hospitalizations	Renal diseases	NA	Time-series Poisson regression with over-dispersion correction (NB when appropriate)	65–84 y: 1.08 (0.97–1.20); >85 y: 1.19 (1.03–1.38)	IRR	Seasonality, long-term trends, over-dispersion
Hopp S. (2018) [30]	Medicare hospitalization claims data	Daily counts	Hospitalizations	All-cause ED	CV, renal, respiratory diseases	Log-linear regression with county fixed effects in matched HW vs. control days	Heat stroke & sunstroke: 22.55 (14.88–34.19); heat exhaustion: 9.38 (6.97–12.63); volume depletion disorder: 1.22 (1.16–1.28); dehydration: 1.21 (1.13–1.30); acute kidney failure, unspecified: 1.15 (1.10–1.20); UTI: 1.11 (1.08–1.15)	RR	County fixed effects, year/month/day of week
Huang Y. S. (2023) [53]	Daily hospital admission counts collected across 23 study sites	Daily counts	Hospitalizations	Renal diseases	NA	Two-stage analysis: conditional quasi-Poisson case-crossover with DLNM + random-effects meta-analysis	1.08 (1.02–1.14)	RR	Year/month/day of week, relative humidity, public holidays, PM_2.5_, O_3_, wind speed
Jung Y. S. (2025) [31]	Medicare fee-for-service inpatient claims data	Individual events	Hospitalizations	All-cause ED	NA	Time-stratified case-crossover with conditional LR and random-effects meta-analysis	Greater Boston Metropolitan Area, GBMA: 1.17 (1.11–1.25); Boston only 1.33 (1.17–1.50)	RR	Seasonality, long-term trends, PM_2.5_, NO_2_, O_3_
Kim H. (2024) [32]	Administrative healthcare claims from Medicare and Medicaid records	Daily counts	Hospitalizations + ED visits	All-cause ED	NA	Poisson regression with fixed effects for ZCTA and calendar time	Heat-related ED visits: 1.10 (1.08–1.12); Heat-related hospitalizations: 1.07 (1.04–1.09)	IRR	ZCTA fixed effects, day of week, public holidays, relative humidity
Knowlton K. (2009) [33]	Hospital discharge data and ED administrative records	Aggregate counts of events	Hospitalizations + ED visits	All-cause ED	Diabetes, CV, neurological, respiratory, renal diseases	Descriptive comparison with chi-square tests	All-cause ED: 1.03 (1.02–1.04); all-cause hospitalizations: 1.01 (1.00–1.02)	RR	NA
Lai E. T. (2025) [54]	Hospital admission and ED records	Daily counts	Hospitalizations	CV, respiratory diseases	NA	Time-stratified case-crossover using conditional Poisson regression	CV admissions: 65–74 y 1.09 (1.01–1.17), >85 1.04 (1.01–1.07); respiratory admissions: 65–74 y 1.10 (1.01–1.20), >85 y 1.04 (1.01–1.08)	RR	Relative humidity, NO_2_, O_3_, PM_10_
Lindstrom S. J. (2013) [43]	Hospital electronic medical records coded using ICD-10	Aggregate counts of events	Hospitalizations + ED visits	All-cause ED	NA	Over-dispersed Poisson regression estimating IRR	ED: 26% in study week vs. 21% in control; total hospital admissions: 33% vs. 30%; general medicine admissions: 88% vs. 81%	Percent (%), descriptive comparisons, *p*-values	Day of week
Liss A. (2017) [34]	Medicare hospitalization claims data	Daily counts	Hospitalizations	All-cause ED	NA	NB GLM	6.89 (4.84–9.80)	RR	Seasonality, public holiday
Liss A. (2019) [35]	MEDPAR inpatient hospitalization records	Daily counts	Hospitalizations	All-cause ED	NA	Harmonic Poisson/NB GLM for seasonal peak estimation	1st seasonal HW: 10.4 (8.5–12.3); 2nd HW: 11.4 (9.6–13.3); 4th HW: 4.78 (3.10–6.46)	RR	Seasonality, climate region
Liu X. (2019) [55]	Hospital information system records from the Mental Health Center of Shandong Province	Individual events	Hospitalizations	Neurological diseases	NA	Conditional LR in time-stratified case-crossover design	Age ≥65 (vs ≤64): 3.03 (1.80–5.13)	OR	Tmean, Tmax, relative humidity, wind speed, atmospheric pressure
Martínez-Solanas È. (2019) [67]	National hospital discharge registry	Daily counts	Hospitalizations	CV, respiratory diseases	NA	Quasi-Poisson regression with DLNM pooled via multivariate meta-analysis	CV admissions: 65–74 y 0.96 (0.93–0.98), 75–84 y 0.99 (0.97–1.00), ≥85 0.98 (0.96–1.00); cerebrovascular admissions: 65–74 y 1.02 (0.98–1.06), 75–84 y 0.97 (0.94–1.00), ≥85 0.97 (0.93–1.02); respiratory admissions: 65–74 y 1.04 (1.01–1.07), 75–84 y 1.06 (1.03–1.08), ≥85 1.09 (1.05–1.13)	RR	Day of week, public holidays, long-term trend, seasonality
Mastrangelo G. (2007) [76]	Regional computerized hospital discharge database	Daily counts	Hospitalizations	All-cause ED	NA	GEE with Poisson distribution and log link	Heat diseases 1.16 (1.12–1.20); respiratory diseases 1.05 (1.03–1.07)	IRR	Day of week
Mayner L. (2010) [44]	Emergency department administrative database and medical record review	Aggregate counts of events	ED visits	All-cause ED	NA	Non-parametric analysis using Kruskal–Wallis tests	ED presentations +19% compared with pre-HW and +16% compared with post-HW	Percent increase (excess risk)	NA
McTavish R. K. (2018) [71]	Administrative healthcare databases	Individual events	Hospitalizations + ED visits	Renal diseases	AKI	Conditional LR	1.11 (1.00–1.23)	OR	NA
Nhung N. T. T. (2023) [64]	Electronic hospital databases from provincial hospitals coded using ICD-10	Daily counts	Hospitalizations	CV, respiratory diseases	NA	Quasi-Poisson GAM time series	Respiratory diseases: Ninh Thuan 1.02 (0.92–1.14), Ca Mau 1.02 (0.91–1.14); CV diseases: 0.92 (0.86–0.99)	RR	Day of week, public holidays, long-term trend, seasonality
Nitschke M. (2011) [45]	Ambulance service call-out records	Daily counts	ED visits	Neurological, respiratory, CV, renal diseases	NA	Poisson case-series regression	HW 2008: hospital admissions: 65–74 y 1.06 (0.82–1.36); 75 y 1.11 (0.90–1.37); HW 2009: hospital admissions 65–74 y 1.08 (0.83–1.40), 75 y 1.14 (0.91–1.42); ED 75 y 1.17 (1.11–1.22)	IRR	NA
Ogbomo A. S. (2017) [24]	Michigan Inpatient Database hospital admission records	Individual events	Hospitalizations	CV, respiratory, renal, metabolic diseases	Diabetes mellitus	Conditional LR	CV diseases: 0.96 (0.91–1.01); respiratory diseases: 1.01 (0.92–1.11); diabetes mellitus: 1.09 (0.85–1.39); renal diseases: 1.20 (0.99–1.45); acute myocardial infarction: 1.06 (0.90–1.26); all natural causes: 0.99 (0.96–1.03)	OR	NA
Parry M. (2019) [46]	Ministry of Health admitted patient data collection system	Individual events	Hospitalizations	CV diseases	NA	Conditional LR	Ischemic heart disease: 0.95 (0.88–1.01); heart failure: 0.96 (0.87–1.06); cardiac arrest: 0.98 (0.70–1.38); heart arrhythmia: 0.99 (0.92–1.07); conduction disorders: 0.88 (0.72–1.09); hypertensive disease: 0.98 (0.79–1.22)	OR	NO_2_, O_3_, PM_10_, public holidays
Phung D. (2016) [62]	Hospital admission records from two hospitals coded using ICD-10	Daily counts	Hospitalizations	CV diseases	NA	Poisson GAM time-series regression	65–84 y: 1.07 (0.86–1.32); 85 y: 0.95 (0.59–1.46)	RR	Seasonality, long-term trends, day of week, public holidays, relative humidity
Ponjoan A. (2017) [68]	Information System for the Development of Research in Primary Care	Individual events	Hospitalizations	Neurological diseases	Stroke	Conditional Poisson regression	0.96 (0.83–1.10)	IRR	Age
Ragettli M. S. (2019) [73]	Swiss Federal Statistical Office Medical Statistics of Hospitals	Daily counts	ED visits	All-cause ED	NA	Stratum-specific quasi-Poisson regression	Jun–Aug 2015: 65–74 y 1.01 (0.99–1.03), ≥75 y 1.05 (1.03–1.06); July 2015 65–74 y 1.04 (1.01–1.07), ≥75 y 1.10 (1.08–1.12)	RR	Long-term trends, seasonality
Santodomingo M. (2024) [36]	California Department of HCAI ED encounter database	Individual events	ED visits	All-cause ED	NA	Conditional LR	HW 95th 1.02 (1.02–1.02); HW 99th 1.04 (1.03–1.05)	OR	NA
Schaffer A. (2012) [47]	ED administrative data systems	Daily counts	ED visits	All-cause ED	NA	Poisson GLM	1.08 (1.04–1.11)	RR	Day of week, year, public holidays
Semenza J.C. (1999) [37]	IHCCCC hospital discharge records coded using ICD-9	Daily counts	Hospitalizations	Metabolic, neurological, kidney diseases	Dehydration, stroke, acute renal failure	Two-tailed t-tests comparing excess vs. expected counts	Excess +838 (vs expected weeks)	Absolute excess count	NA
Smith S. (2016) [77]	EDSSS	Aggregated population-level morbidity measures	ED visits	CV diseases	Myocardial ischemia	NA	65–74 y 0.88 (0.46–1.29); >75 y 0.75 (0.65–0.84)	IRR	NA
Sohail H. (2020) [78]	National hospital discharge register	Daily counts	Hospitalizations	Respiratory diseases	NA	Multivariate Poisson time-series regression	1.40 (1.09–1.80)	RR	Year/month/day of week, public holidays, relative humidity, NO_2_, O_3_, PM_10_, PM_2.5_
Toloo G.S. (2014) [48]	Queensland Health Information Centre administrative hospital and mortality records	Daily counts	ED visits	All-cause ED	NA	Over-dispersed Poisson GAM	HW1 (≥34 °C): 65–74 y 7.29 (3.93–13.53); 75 y 9.17 (5.45–15.44). HW2 (≥37 °C): 65–74 y 23.54 (8.99–61.69); 75 y 37.55 (18.34–76.86)	RR	Relative humidity, O_3_, PM_10_, NO_2_, day of week
Tong S. (2014) [49]	Queensland Health and Queensland Treasury hospitalization and mortality records	Daily city-aggregated counts	ED visits	All-cause ED	NA	Over-dispersed Poisson GAM	65–74 y 1.08 (1.01–1.15); ≥75 y 1.21 (1.16–1.26)	RR	Day of week, relative humidity, PM_10_, NO_2_, O_3_
Trang P.M.(2016) [63]	Hospital electronic registry records with diagnoses coded by mental health specialists using ICD-10	Daily counts	Hospitalizations	Neurological diseases	NA	Zero-inflated NB regression with seasonal and temporal adjustment	3.20 (1.63–6.29)	RR	Seasonality, long-term trend, day of week
Wang Y. (2016) [38]	County-level daily hospital admission counts from U.S. Medicare inpatient administrative claims	Daily counts	Hospitalizations	Neurological diseases	stroke	Random-effects Poisson regression in time-stratified case-crossover design	65–74 y 9.5 (6.9–17.0); 75–84 y 11.0 (6.9–14.7); >84 y 13.1 (7.5–19.0)	OR	Day of week, public holidays
Wilson L.A. (2013) [50]	New South Wales statewide death registry	Individual events	Hospitalizations	Metabolic, respiratory, neurological, kidney disease	Diabetes, fluid/electrolyte disorders, COPD urolithiasis, dementia	Conditional LR	1.05 (1.03–1.07)	OR	Public holiday
Yin J. (2025) [56]	Daily counts of ischemic stroke hospital admissions	Daily counts	Hospitalizations	Neurological diseases	Stroke	DLNM with quasi-Poisson regression (GAM framework)	1.20 (1.05–1.36)	RR	Relative humidity, PM_2.5_, NO_2_, O_3_
Yong K.H. (2023) [57]	Hospital admission records coded using ICD-10	Daily counts	Hospitalizations	All-cause ED	NA	Generalized additive model (GAM) with distributed lag model (DLM)	1.23	RR	Day of week, long-term trend, relative humidity, wind speed
Zhang A. (2019) [59]	Hospital electronic medical records for outpatient visits coded using ICD-10	Individual events	Outpatient care	Respiratory diseases	NA	Conditional Poisson regression in time-stratified case-crossover design	1.41 (1.11–1.79)	RR	Rainfall, wind speed, relative humidity
Zhang H. (2024) [58]	Outpatient diagnoses coded using ICD-10	Daily counts	Outpatient care	Neurological diseases	NA	Quasi-Poisson GLM with DLM	1.32 (1.09–1.55)	RR	Relative humidity, PM_2.5_, day of week
Zhang K. (2015) [39]	Mortality records coded using ICD-10 and ED visit records coded using ICD-9	Daily counts	ED visits	All-cause ED	NA	DLNM with over-dispersed Poisson regression	1.13 (1.06–1.20)	RR	Day of week, long-term trend, seasonality

AKI = Acute Kidney Injury; CER = Cumulative Excess Risk; CI = Confidence Intervals; CM = Clinical Modification; COPD = Chronic Obstructive Pulmonary Disease; CV = Cardiovascular; DID = Difference-in-Differences; DLNM = Distributed Lag Non-Linear Model; DLM = Distributed Lag Model; ED = Emergency Department; EDSSS = Emergency Department Syndromic Surveillance System; EHF = Excess Heat Factor; GAM = Generalized Additive Model; GBMA = Greater Boston Metropolitan Area; GEE = Generalized Estimating Equations; GLM = Generalized Linear Model; HCAI = Health Care Access and Information; HRI = Heat-Related Illness; HW = Heat Wave; ICD = International Classification of Diseases; IHCCCC = Illinois Health Care Cost Containment Council; INE = Spanish National Institute of Statistics; IRR = Incidence Rate Ratio; ISAAC = Integrated South Australian Activity Collection; LR = Logistic Regression; MEDPAR = Medicare Provider Analysis and Review; NB = Negative Binomial; NO_2_ = Nitrogen Dioxide; O_3_ = Ozone; OR = Odds Ratio; PM_10_ = Particulate Matter ≤ 10 µm; PM_2.5_ = Particulate Matter ≤ 2.5 µm; RR = Relative Risk; Tmax = Daily Maximum Temperature; Tmean = Daily Mean Temperature; Tmin = Daily Minimum Temperature; UTI = Urinary Tract Infection; ZCTA = Zip Code Tabulation Area.

## Data Availability

The data extracted from the included studies and the materials used for the review are available within the manuscript and its Supplementary Materials. Additional extracted data and review materials are available from the corresponding authors upon reasonable request.

## Supplementary Materials

The following supporting information can be downloaded at https://www.mdpi.com/article/10.3390/diseases14050176/s1: Supplementary Table S1: Full search strings used in PubMed, Scopus, and Web of Science, including all controlled vocabulary terms and free-text keywords, reported exactly as executed; Supplementary Figure S1: (a) A forest plot and (b) funnel plot of the fixed-effects model assessing hospitalizations; Supplementary Figure S2: (a) A forest plot and (b) funnel plot of the fixed-effects model assessing emergency department visits; Supplementary Figure S3: A forest plot and (b) funnel plot of the fixed-effects model assessing studies reporting odds ratios; Supplementary Figure S4: (a) A forest plot and (b) funnel plot of the fixed-effects model assessing studies reporting risk ratios or incidence rate ratios.

## Abbreviations

The following abbreviations are used in this manuscript:

DLM	Distributed Lag Model
DLNM	Distributed Lag Non-Linear Model
ED	Emergency Department
EHF	Excess Heat Factor
UNDRR	United Nations Office for Disaster Risk Reduction
WMO	World Meteorological Organization
