# Supplementary material for: Effects of Heat Waves on Hospitalizations, Emergency Department Visits, and Outpatient Care in Frail Older Adults: A Systematic Review and Meta-Analysis
Source: Diseases. 2026 May 18;14(5):176. doi: 10.3390/diseases14050176 (PMC13205340; doi:10.3390/diseases14050176)

Effects of heat waves on hospitalizations, emergency department visits, and outpatient care in frail older adults: a systematic review and meta-analysis

Table S1. Full search strings used in PubMed, Embase, and Scopus, including all controlled vocabulary terms and free-text keywords, reported exactly as executed.

Database	Search strategy	Number of identified records
PUBMED	("Climate Change"[MeSH Terms] OR "Global Warming"[MeSH Terms] OR "Extreme Weather"[MeSH Terms] OR "Climate Change"[All Fields] OR "climate changes"[All Fields] OR "heat waves"[All Fields] OR "heat wave"[All Fields] OR "Extreme Heat"[MeSH Terms] OR "Extreme Heat"[All Fields] OR "extreme heats"[All Fields] OR "Extreme Weather"[All Fields] OR "climate events"[All Fields] OR "climate event"[All Fields] OR "heat stress"[All Fields] OR "environmental change"[All Fields] OR "environmental changes"[All Fields] OR "Global Warming"[All Fields]) AND ("Hospitalization"[MeSH Terms] OR "emergency service, hospital"[MeSH Terms] OR "Outpatients"[MeSH Terms] OR "Emergency Service"[All Fields] OR "Ambulatory Care"[MeSH Terms] OR "Hospitals"[MeSH Terms] OR "hospitalization*"[All Fields] OR "emergency room"[All Fields] OR "outpatient care"[All Fields] OR "healthcare utilization"[All Fields]) AND ("Frail Elderly"[MeSH Terms] OR "frail"[All Fields] OR "frails"[All Fields] OR "frailty"[MeSH Terms] OR "frailty"[All Fields] OR "frailness"[All Fields] OR "frailty"[MeSH Terms] OR "frailty"[All Fields] OR "frailties"[All Fields] OR "Aged"[MeSH Terms] OR "Geriatrics"[MeSH Terms] OR "elderly"[All Fields] OR "older adults"[All Fields] OR "Aged"[All Fields] OR "geriatric"[All Fields] OR "senior"[All Fields] OR "aging population"[All Fields])	616
SCOPUS	("Extreme Weather" OR "climate change" OR "heat wave" OR "extreme heat" OR "climate event" OR "heat stress" OR "environmental change" OR "global warming") AND (hospital OR "emergency room" OR “Emergency Service“ OR "outpatient care" OR "healthcare utilization") AND (frail OR "elderly" OR "older adults" OR "aged" OR "geriatric" OR "senior" OR "aging population")	1,153
WEB OF SCIENCE	ALL=(("Extreme Weather" OR "climate change" OR "heat wave" OR "extreme heat" OR "climate event" OR "heat stress" OR "environmental change" OR "global warming") AND (hospital OR "emergency room" OR “Emergency Service“ OR "outpatient care" OR "healthcare utilization") AND (“frail” OR "elderly" OR "older adults" OR "aged" OR "geriatric" OR "senior" OR "aging population"))	1,050

Figure S1. (a) A forest plot and (b) funnel plot of the fixed-effects model assessing hospitalizations.

a)

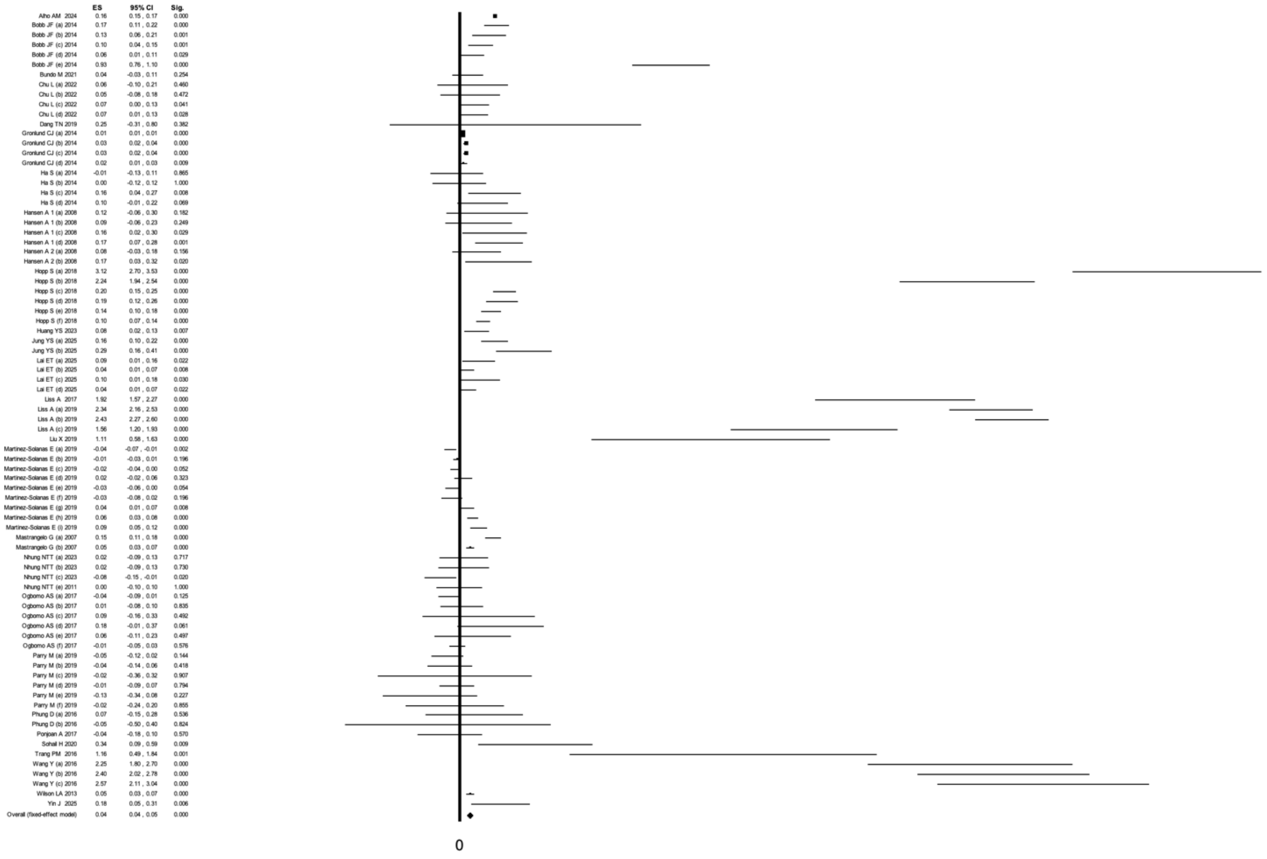

b)

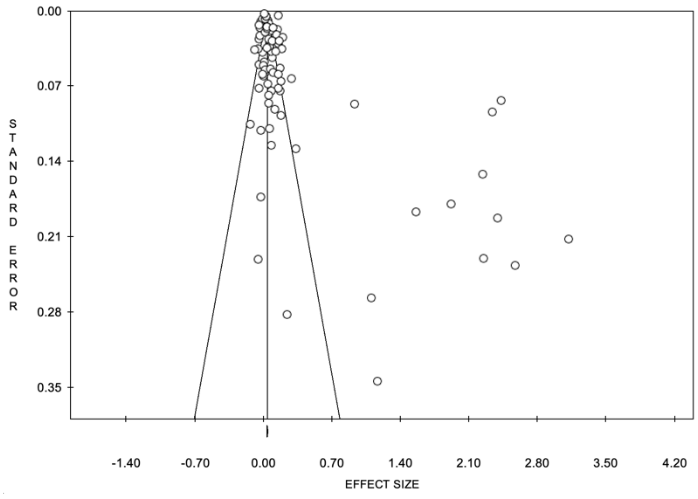

Figure S2. (a) A forest plot and (b) funnel plot of the fixed-effects model assessing emergency department visits.

a)

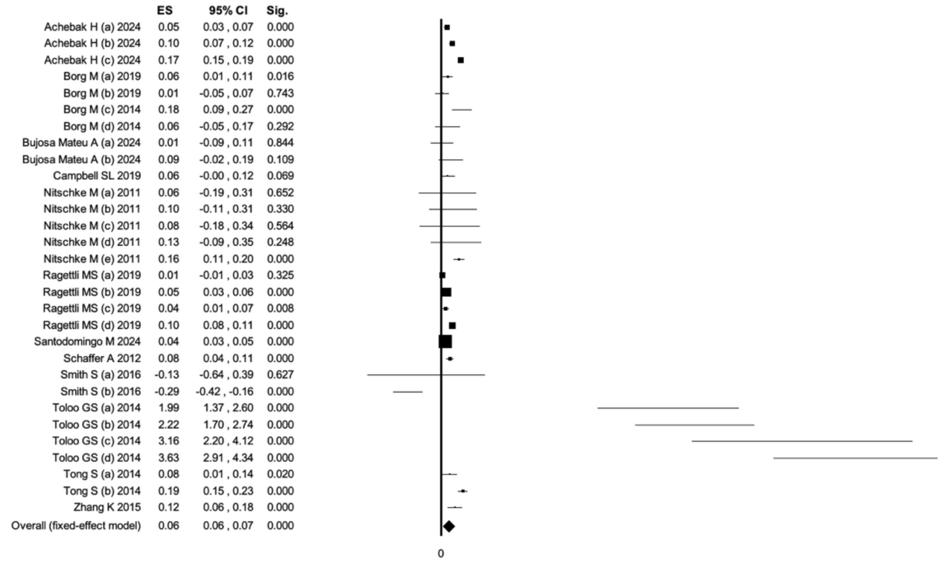

b)

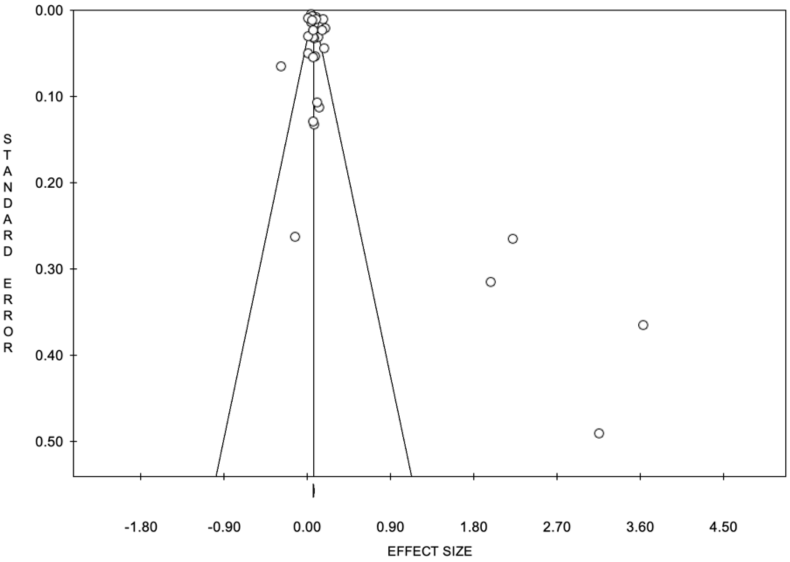

Figure S3. (a) A forest plot and (b) funnel plot of the fixed-effects model assessing studies reporting odds ratios.

a)

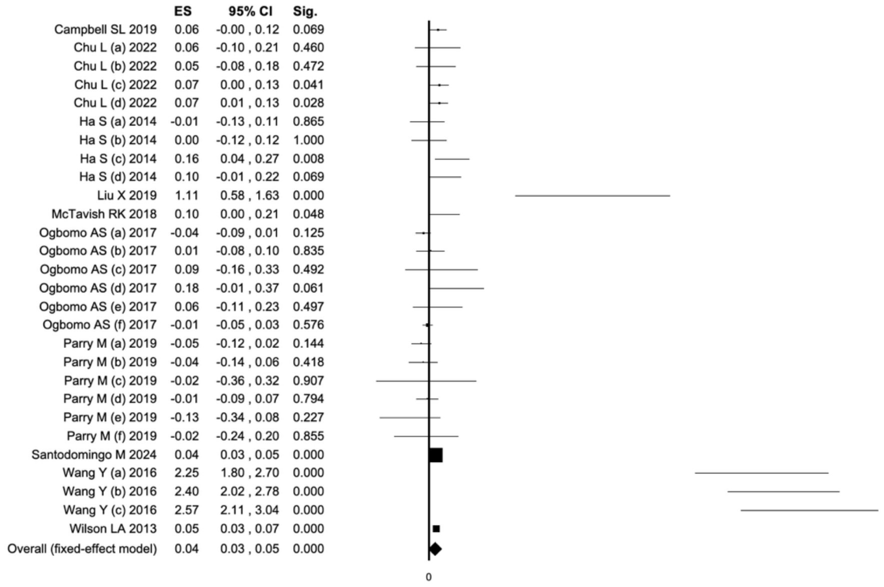

b)

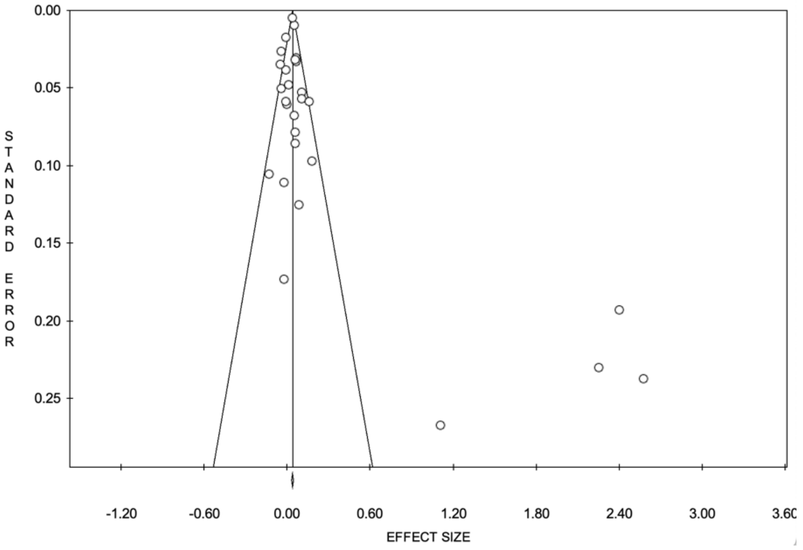

Figure S4. (a) A forest plot and (b) funnel plot of the fixed-effects model assessing studies reporting risk ratios or incidence rate ratios.

a)

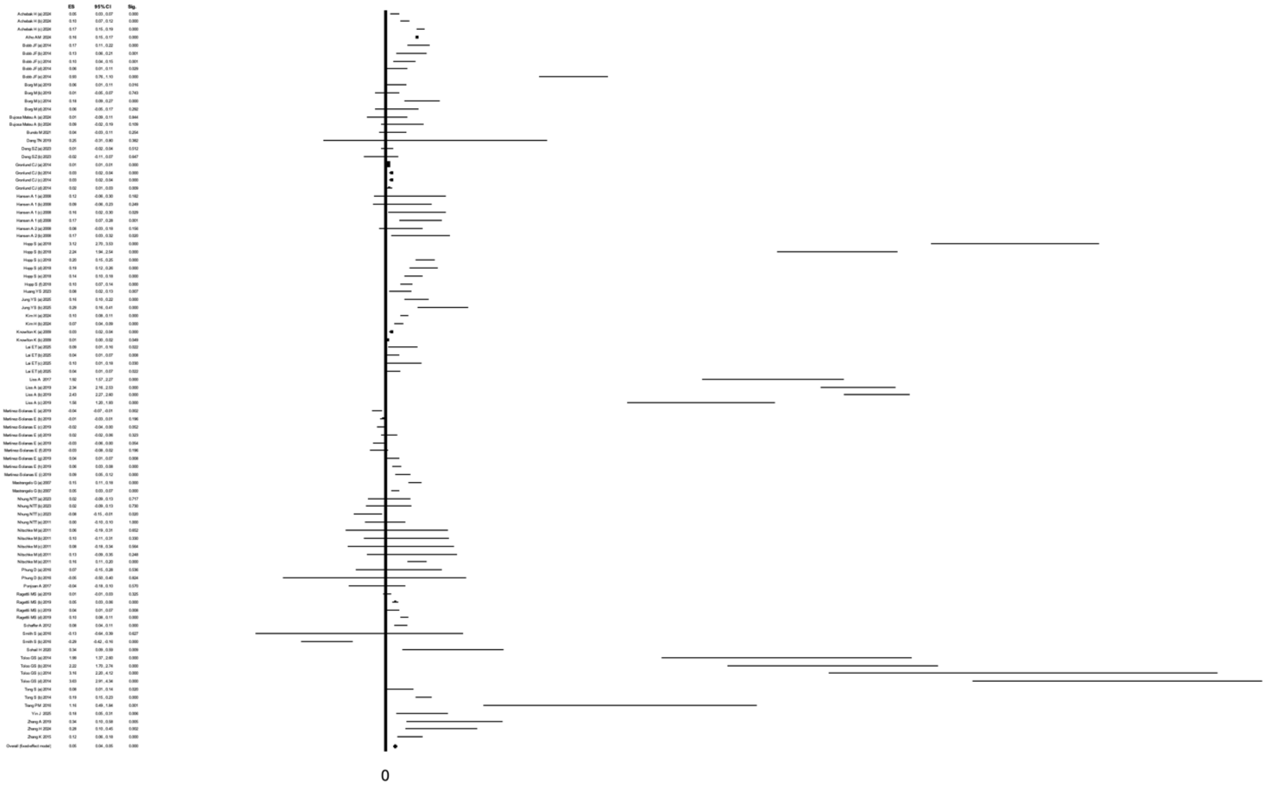

b)

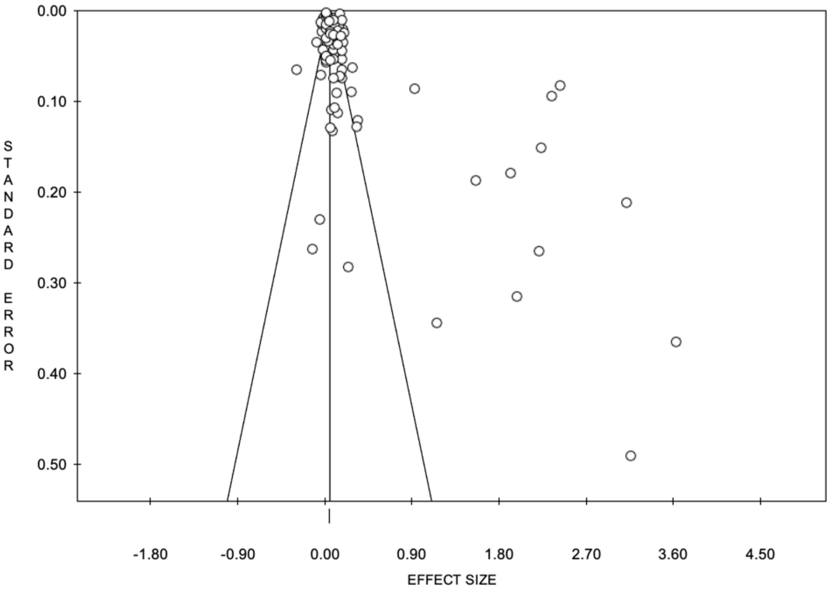

Supplement: Supplementary file 1 [file diseases-14-00176-s001.zip › diseases-4262669-supplementary.pdf]
